# Synthesis of N-doped ZnO nanoparticles with cabbage morphology as a catalyst for the efficient photocatalytic degradation of methylene blue under UV and visible light

**DOI:** 10.1039/c8ra09962f

**Published:** 2019-03-06

**Authors:** Eswaran Prabakaran, Kriveshini Pillay

**Affiliations:** Department of Applied Chemistry, University of Johannesburg Johannesburg South Africa kriveshinip@uj.ac.za +27 11 5596425 +27 11 5596128

## Abstract

In this study, the synthesis of nitrogen-doped zinc oxide nanoparticles with a cabbage like morphology (N-ZnONCBs) by a hydrothermal method using zinc acetate dihydrate as a precursor and hydrazine monohydrate as a nitrogen source is reported. N-ZnONCB were characterized using UV-visible Spectroscopy (UV-Vis), Fluorescence Spectroscopy, Fourier Transmittance Infrared Spectroscopy (FTIR), X-ray diffraction (XRD), Raman Spectroscopy, Thermogravimetric Analysis (TGA), Scanning Electron Microscopy (SEM), Transmission Electron Microscopy (TEM), Electron Dispersive Spectroscopy (EDS) and EDX elemental mapping. N-ZnONCBs were tested for their photocatalytic capabilities in the degradation of methylene blue (MB) under UV-light and visible light irradiation for about 0 to 80 minutes and 0 to 50 min respectively. The N-ZnONCB catalyst demonstrated improved photodegradation efficiency (98.6% and 96.2%) and kinetic degradation rates of MB (*k* = −0.0579 min^−1^ and *k* = −0.0585 min^−1^) under UV light and visible light irradiation at different time intervals. The photodegradation study was also evaluated with different dosages of N-ZnONCB catalyst, different initial concentrations of MB and variation in the pH (3, 5, 9 and 11) of the solution of MB under UV light and visible light irradiation. The photocatalytic degradation intermediate products were obtained by liquid chromatography mass spectra (LC-MS) and also complete mineralization was determined by using Total Organic Carbon (TOC) studies. This photocatalyst was also tested with 2,4-dichlorophenol (2,4-DCP) under visible light irradiation at different time intervals. Fluorescence and quenching studies were performed for the binding interaction between the N-ZnONCB catalyst and MB dye. A Zetasizer was used to find the charge and average size of the N-ZnONCB catalyst and also the charge of the N-ZnONCB catalyst before and after MB dye solution adsorption. The N-ZnONCB catalyst was also tested for its photostability and reusability with a percentage degradation rate of MB (93.2%) after 4 cycle experiments. These results have clearly demonstrated that the N-ZnONCB catalyst can be applied for the photocatalytic degradation of MB from wastewater samples.

## Abstract 2

In this study, the synthesis of nitrogen-doped zinc oxide nanoparticles with a cabbage like morphology (N-ZnONCBs) by a hydrothermal method using zinc acetate dihydrate as a precursor and hydrazine monohydrate as a nitrogen source is reported.

## Graphical abstract

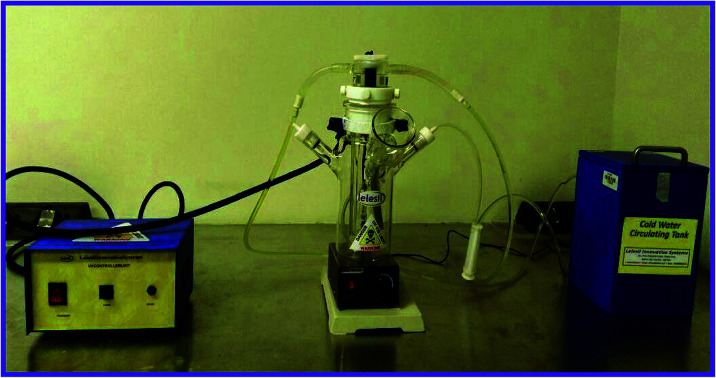

## 1. Introduction

Developing countries need to advance their financial growth by increased industrial activity due to their increase in population. Such industries are the fundamental cause of water contamination. Industries such as the textile industry produce organic dyes and these dyes can no longer be removed by biodegradable methods without difficulty. Such dyes can be poisonous to aquatic organisms, plants and the human body. The removal of organic pollutants has been carried out with different processes, in which photocatalytic methods have been powerful and simple for the degradation of organic dyes.^1,2^ Specifically, semiconductor metallic oxide nanomaterials have been used to remove organic dyes from aqueous solution.^3^ Metal oxides have exhibited good photocatalytic activities under sunlight and have been used to degrade poisonous organic dyes to non-poisonous forms.^4^ Recently, the degradation of pollutants was carried out with unique semiconductor metal oxide nanoparticles which include TiO_2_, ZnO, WO_3_, In_2_O_3_ and SnO_2_.^5–9^ Among these, ZnO showed superior properties, including an absorption range within the visible region, high photo-stability, good sensor abilities, light-emitting diodes, and solar light harvesting.^10–14^ The photocatalytic performance of ZnO was not effective for the degradation of organic dyes due to an extensive band gap (3.37 eV) and the recombination of electron–hole pairs.^15^ In the past decade, researchers have synthesized ZnO nanoparticles with different morphologies such as nanorods, nanobelts, nanoarrows, nanoplates, nanotubes, nanowires, nanoflowers, and nanospheres for photocatalytic applications.^16–24^ In addition, functionalized ZnO nanoparticles had been proven to have distinctive properties which include biocompatibility, a non-toxic nature, highly stability and good photo-catalytic potential.^25–28^

Doping and introducing impurities in ZnO nanoparticles have rendered them more photocatalytically active over a wide range of wavelengths (UV region to visible region) by reducing the bandgap energy.^29^ ZnO nanoparticles have been modified with different dopants consisting of N and S atoms for enhancing the photocatalytic oxidation of organic dyes under UV light irradiation.^30,31^ Nitrogen-doped ZnO nanoparticles in the form of nanorods and nanowires have been used for water splitting applications.^32^ Nowadays, N-doping on ZnO nanoparticles has been conducted using various methods such as thermal evaporation,^32^ pyrolysis^33^ and thermal nitridation^34^ with ammonia as a nitrogen source precursor. The methods have however demonstrated a few disadvantages like higher temperature demands and these materials cannot be used in photocatalytic applications. Hydrothermal methods have exhibited better advantages such as simple processes, lower temperature demands, eco-friendliness and better time control. This method was also used to prepare different morphologies of ZnO nanoparticles such as nanorods, nanotowers, nanotubes, nanoflowers and nanovolcanoes as reported previously.^35^ The size-controlling agent ethylenediamine was applied for the synthesis of various sizes of ZnO nanoparticles like nanorods and nanoflowers with hydrothermal methods at low temperatures.^36^

Dye industries have been releasing considerable amounts of dye wastewater into the environment and as an end result this can affect the aquatic systems and result in soil deterioration.^37,38^ Among them, methylene blue (MB) is a cationic dye consisting of a conjugated aromatic moiety which makes it favorable for photocatalytic activities because of easy identification and color change. Moreover, it is relatively easy to identify the intermediate products during photocatalytic degradation.^39^ The identification of intermediates is in turn important for elucidating the degradation mechanism on the aromatic group and methyl functionalized on benzene ring.^40–42^ Hence, MB degradation processes are crucial for noting whether complete mineralization occurs during photocatalysis.

Chlorophenol derivatives are highly toxic pollutants in terms of acting as carcinogens and contaminating the ecosystem. These toxic compounds have been used for the manufacturing of pesticides, dyes, drugs, papers and plastics.^43,44^ Consequently, they have induced various problems to environmental and aquatic structures because of excessive toxicity, bioaccumulation, intense odor and recalcitrance.^45^ The limitation of chlorophenols was listed by the US Environmental Protection Agency due to the destruction of soil and water systems.^46^ Among these, 2,4-DCP has been identified as a priority pollutant due to its highly toxic nature. At the same time 2,4-DCP has also been used in the manufacture of fertilizers, herbicides and pesticides.^47^ Despite the fact that 2,4-DCP is continuing to contaminate water that is released into the environment, it is still extensively used industrially. Therefore, MB and 2,4-DCP have been chosen as model pollutants for photocatalytic applications.

In this study, synthesis of N-ZnO nanoparticles with cabbage morphology using a hydrothermal method is therefore reported. N-ZnONCBs was prepared using Zn(Ac)_2_·2H_2_O, hydrazine monohydrate as precursors and NaOH was used for adjusting the pH to 8.0. Hydrazine monohydrate supplied the nitrogen source as an NH_3_ dopant and subsequently ZnONCBs were formed. N-ZnONCBs was used as photocatalysts for the degradation of MB and under UV light and visible light irradiation for about 0 min to 80 minutes and 0 to 50 minutes, respectively. The photocatalytic efficiency was tested for the degradation of MB with different dosages of N-ZnONCBs catalyst, different initial concentrations of MB and different pH of MB solution under UV light and visible light irradiation. The photocatalytic ability and rate of degradation of MB using N-ZnONCBs as photocatalysts was compared with those achieved with other morphologies of ZnO nanoparticles as photocatalysts.

## 2. Experimental section

### 2.1. Chemicals and reagents

Zinc acetate dihydrates (Zn (CH_3_COO)_2_·2H_2_O), hydrazine monohydrate (NH_2_–NH_2_·H_2_O), 2,4-dichlorophenol and sodium hydroxide (NaOH) were purchased from Sigma-Aldrich. Methylene blue, ethanol and KBr were obtained from Merck, South Africa. Ultrapure de-ionized water (resistivity > 18.5 MΩ cm) was used to prepare the working solutions and this is obtained from water purification systems (Milli-Q, Millipore). All solvents and other chemicals were purchased from Sigma-Aldrich and were of reagent grade.

### 2.2. Synthesis of N-ZnONCBs catalyst

**Scheme 1:**
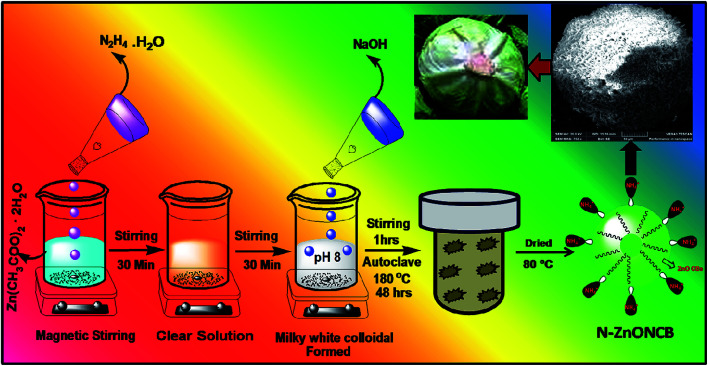
Synthesis of N-ZnONCBs catalyst by hydrothermal method.

1 g (1.5 M) of zinc acetate dehydrate was dissolved in 50 ml of water while stirring for 30 minutes and 5 ml of hydrazine monohydrate solution was introduced to obtain a clear solution under constant stirring again for 30 minutes. 1.2 g (3 M) of NaOH solution was slowly added drop wise into the mixture of solution to form a white precipitate at pH 8. The white precipitate was dissolved for 1 h under stirring to obtain a homogeneous solution. The solution was then transferred into a 100 ml Teflon coated autoclave and heated at 180 °C in the muffle furnace for 48 h. After the autoclave was allowed to cool to room temperature a white granular solid representing the N-ZnONCBs catalyst formed at the bottom of the Teflon cup.^48^ The white granular solid was washed with double distilled water and ethanol several times and dried in an oven at 80 °C for 12 h. A schematic diagram of this procedure is shown in Scheme 1.

### 2.3. Characterization techniques

The solution form of the N-ZnONCBs catalyst was tested for its optical properties using a Schimadzu UV-1208 model UV-visible spectrophotometer (Japan). Photoluminescence properties of N-ZnONCBs catalyst were investigated with the aid of a fluorescence instrument (PerkinElmer Spectrum spectrometer). The formation of N-ZnONCBs catalyst and its functional groups and bond formation were confirmed by a Perkin-Elmer PE1600 FTIR spectrophotometer (USA) within the range of 4000–400 cm^−1^ and KBr which was used to make the pellet. The crystalline and approximate size of N-ZnONCBs catalyst was calculated by using a X-ray diffraction (Panalytical X-PertPro X-ray Diffractometer with Philips PW1729 diffractometer with working systems of Cu Kα radiation (*λ* = 1.5406 Å) operating at 45 kV and 40 mA). The vibration mode of the molecules of N-ZnONCBs catalyst was determined by a Raman scattering method (Perkin Elmer spectrum spectrometers at a laser excitation line of 532 nm). The morphology of N-ZnONCBs catalyst and element mapping were investigated by using Scanning Electron Microscope (TESCAN, VEGA SEM) with the working system of electron acceleration voltage 20 kV with carbon coating for excellent images of the N-ZnONCBs catalyst. The size of the N-ZnONCBs catalyst was measured by a Transmission electron microscope (TEM JEOL JEM-2100F) with electron accelerating voltage of 90 kV. Zetasizer measurements were conducted to determine the charge and average size of the nanoparticles with Malvern Instruments Ltd., GB. Before analysis the sample was dispersed in water (10 ml, 10 mg) with sonication for 15 minutes. The BET technique was used to determine the surface area and pore size distribution curve of the prepared material by nitrogen adsorption–desorption measurements using a Micrometrics, ASAP 2020, surface area and porosity analyzer. The pH measurements were conducted with an OHAUS starter 2100 (USA) pH probe. The photocatalytic degradation of MB solution was conducted in a PROTEA LABS, Photochemical Reactor 500 ml capacity with UV lamp (250 W medium pressure) and visible lamp (250 W high pressure) (India).

### 2.4. Photocatalytic instrument set up

**Fig. 1:**
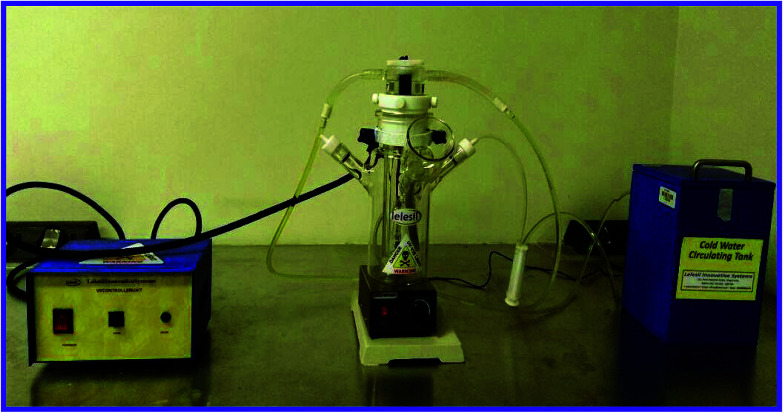
Setup of photoreactor for photocatalytic degradation application.

The photocatalytic reactor was purchased from India. This reactor consists of a double layer of borosilicate glass, wherein the internal glass is connected to the water cooling system to control UV light which produces heat. The outer layer of borosilicate glass brings the MB dye solution and the N-ZnONCBs catalyst into contact for the photocatalytic reaction. A 250 W UV light was used to supply the AC current of 230 V for photocatalytic degradation MB dye solution. Some points of the photocatalytic response were carried with regular stirring for appropriate catalytic response at different times. The photocatalyst reactor set up image is shown in Fig. 1.

### 2.5. Photocatalytic degradation experiment

The photocatalytic reaction of MB was conducted in a photocatalytic reactor and its cylindrical glass was made with borosilicate with 500 ml capacity. The UV lamp (250 nm) was placed in the center of the cylindrical glass. The outer layer of glass in cylindrical vessels was used to circulate of water to control the excess of heat. 15 ppm (3.75 mg L^−1^) of MB was dissolved in 400 ml water and accompanied by the addition of 100 mg (250 mg L^−1^) N-ZnONCBs catalyst with stirring for 30 minutes under dark conditions before starting the photocatalysis under UV irradiation. The samples were collected at each time interval of 10 to 90 minutes under UV light irradiation. The PVDF filter was used to separate the N-ZnONCBs catalyst and the solution of MB concentrations were analysed with UV-visible spectroscopy. The absorption of the MB peak was observed at 665 nm and photocatalytic degradation efficiency *η*(%) was determined using following eqn (1).1
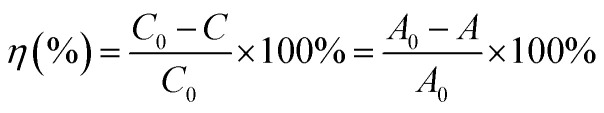
where *C*_0_ is the absorption of the initial concentration of MB dye, *C* is the concentration after an irradiation time of UV light, *A*_0_ is the initial absorbance of the MB solution and *A* is the final absorbance of the MB solution at 665 nm. The total organic carbon (TOC) was investigated by using a Shimadzu (model TOC-2000) TOC technique.

### 2.6. Liquid chromatography-mass spectrometry (LC-MS) study

Liquid chromatography-mass spectrometry (LC-MS) was carried by using liquid chromatography with a binary solvent gradient pump and injector automatic (Shimadzu LCMS-M, Japan). 15 ppm of MB dye degraded products had been separated with a VP-ODS column 150 × 20 mm and particles size 4.6 μm. The UV detector site at 254 nm and also the signal was obtained by using lab solution software. The mobile phases were 0.01 M ammonium acetate, acetic acid at pH 5.3 and acetonitrile with elution for 10% to 90% for 32 min, the flow rate for 0.4 mL min^−1^ and injector volume for 2 μL. LC-MS was set by using electrospray ionization with the polarity of positive and negative working system.

### 2.7. Fluorescence and quenching study

The fluorescence and quenching activity was determined with the N-ZnONCBs catalyst, MB dye and 2,4-DCP. The fluorescence and quenching analyses were investigated by using 1 × 10^−6^ M of (MB and 2,4-DCP) and (0.1 × 10^−6^ M to 0.6 × 10^−6^ M) of N-ZnONCBs catalyst. The quenching behavior was evaluated by a fluorescence spectrometer at an excitation wavelength of 630 nm for MB and 230 nm for 2,4-DCP and an emission wavelength at 680 nm for MB and 350 nm for 2,4-DCP. Therefore, the fluorescence quenching was confirmed by use of a fluorophotometer with different concentrations of quencher and N-ZnONCBs catalyst and the same concentrations of MB and 2,4-DCP. The fluorescence intensity was noted by the binding interaction among the N-ZnONCBs catalyst, MB and 2,4-DCP.

### 2.8. Total organic carbon (TOC) study

The total organic carbon (TOC) was determined by using a TOC analyser (Teledyne Tekmar Introduces Fusion Total Organic Carbon (TOC), United States). It was used to determine the percentage of removal of carbon content during the photocatalytic degradation of MB dye under the UV light and visible light irradiation at various time intervals.

## 3. Result and discussion

### 3.1. Mechanism of N-ZnONCBs catalyst formation

**Scheme 2:**
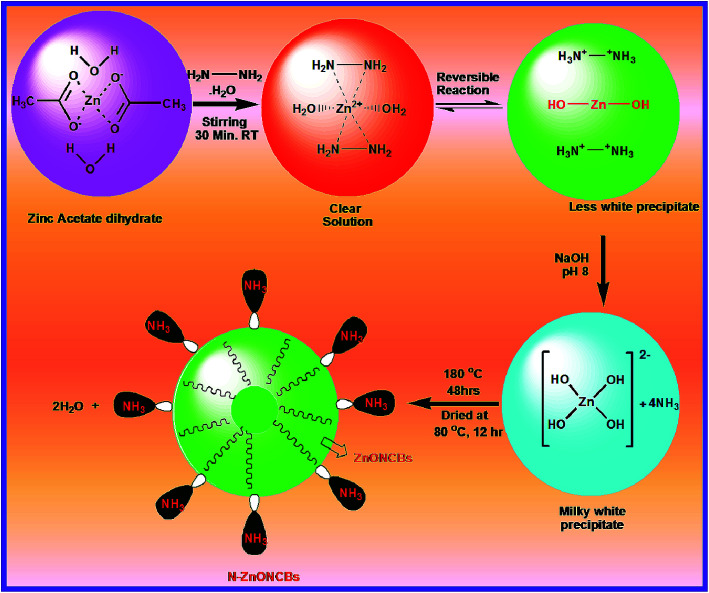
Mechanism for the synthesis of N-ZnONCBs catalyst.

N-ZnOCBs catalyst was synthesized by the use of hydrazine hydrate as an electron donor and it easily coordinates with metallic (Zn^2+^) ions. It produced ammonia as a weak base during the hydrolysis of zinc acetate dehydrates.^49^ The amine group of hydrazine hydrate reacted with Zn^2+^ ions to provide the complex of [Zn(N_2_H_4_)_2_]^2+^ forming a clean transparent solution.^50^ This process is a reversible process, N_2_H_4_ reacts with water molecules to give (H–NH_2_–NH_2_H)^2+^ and 2 OH^−^. Two molecules of OH^–^ ions react with Zn^2+^ ions to form Zn(OH)_2_. Then the addition of NaOH produced a milky white colloidal precipitate to form [Zn(OH)_4_]^2−^.^51^ In addition, [Zn(OH)_4_]^2−^ was heated at 180 °C for 48 h to obtain ZnONCBs and NH_3_ complexed and the elimination of the water molecule. The formation of N-ZnONCBs catalyst has exhibited an absorption spectrum in the visible region and the nitrogen peak of NH_3_ has an absorption spectrum in the UV region.^52^ The detailed mechanism of N-ZnONCBs catalyst formation is shown in Scheme 2.

### 3.2. FT-IR characterization

**Fig. 2:**
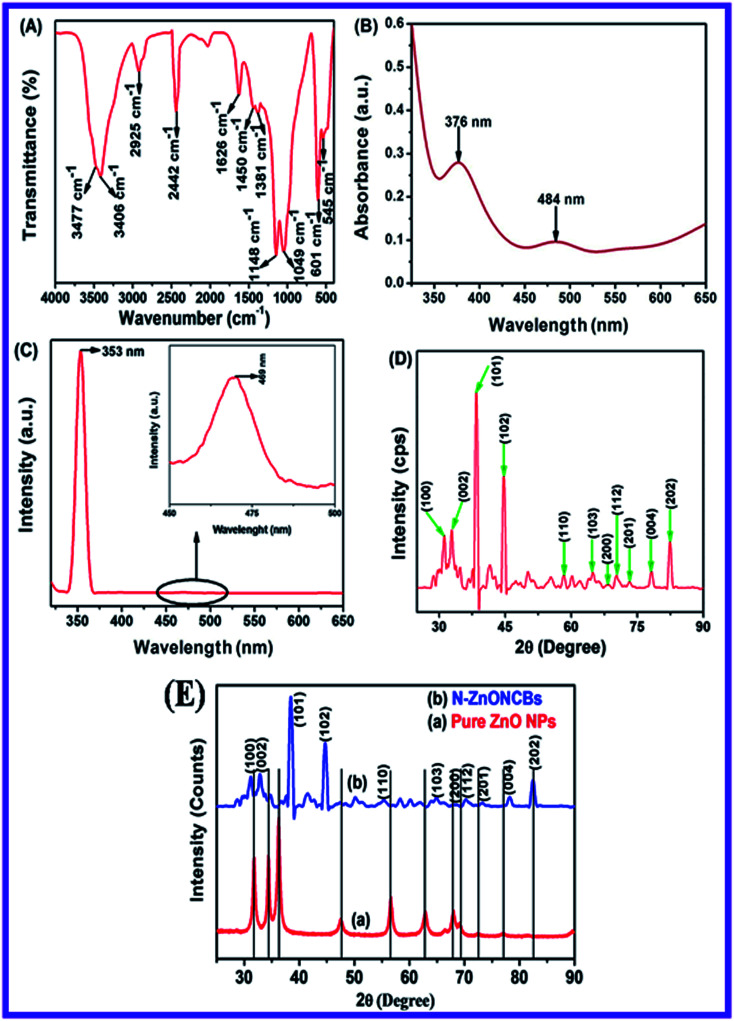
(A) FT-IR spectrum of N-ZnONCBs, (B) DRS absorption spectrum of the N-ZnONCBs, (C) fluorescence spectrum of N-ZnONCBs, (D) X-ray diffraction spectrum of N-ZnONCBs and (E) comparison of X-ray pattern of (a) pure ZnONPs and (b) N-ZnONCBs catalyst.

The functional groups of the N-ZnONCBs catalyst were investigated with FT-IR spectroscopy as shown in Fig. 2(A). Two kinds of peaks have been discovered, a sharp peak at 3477 cm^−1^, which was assigned to the O–H stretching vibration of the water moiety and the shortest peak at 3406 cm^−1^ due to the N–H stretching of the hydrazine molecule.^53^ The peak around 2925 cm^−1^ can be assigned to the asymmetric vibration of C–H group. The peaks at 1381 cm^−1^ and 1450 cm^−1^ are the Zn–N bond and N–Zn–O bond stretching vibrations and these vibrations provided evidence the successful formation of N-ZnONCBs catalyst.^54^ An extended sharp peak which split into two small sharp peaks at 1148 cm^−1^ and 1049 cm^−1^, were assigned to the symmetric stretching vibration of O–C=O and –C–O groups respectively. The peak which appeared at 2442 cm^−1^ was due to the asymmetric stretching vibration of O–C=O group.^55^ The peaks at 1626 cm^−1^ have exhibited the symmetric stretching vibration of –C=O group. The main observation is the Zn–O bond in N-ZnONCBs catalyst whose stretching was observed at 601 cm^−1^ and 545 cm^−1^.^56^ All of the above observations confirmed the formation of the N-ZnONCBs catalyst.

### 3.3. Optical characterizations of N-ZnONCBs catalyst

The optical study of the N-ZnONCBs catalyst was investigated by using a UV-visible diffuse reflectance spectroscopy (DRS) as shown in Fig. 2(B). Fig. 2(B) indicates that two types of peaks have been observed in the UV and visible regions with corresponding wavelengths 376 nm and 484 nm, respectively. The peak at 376 nm clearly demonstrated a slight extension into the visible region due to N-doped ZnONCBs catalysts with a few disordered states of Zn interstitial or O_2_ vacancy atoms which also extended into visible region of absorption.^57^ The band gap energy (*E*_g_ = 3.29 eV) of N-ZnONCBs was also decreased because of N-doping on the ZnONCBs catalyst.^58^ The small broad peak observed within the visible region at 484 nm also confirmed N-doping on the ZnONCBs catalyst.^59^

The emission spectra of N-ZnONCBs catalyst were also explored and the two peaks which include a sharp peak at 353 nm and a weak broad visible emission at 469 nm were observed as shown in Fig. 2(C). The sharp peak occurred due to free excitons recombination and near-band-Edge UV emission.^60^ The weak broad visible emission peak was formed because of some extrinsic or intrinsic defects and is clearly shown in Fig. 2(C) (inset).^61^ These emission peaks confirmed that N-ZnONCBs catalyst had good optical properties.

### 3.4. X-ray diffraction characterization

The X-ray diffraction spectrum of N-ZnONCBs catalyst revealed crystallinity of materials as shown in Fig. 2(D). The diffraction peaks observed 31.65°, 34.35°, 36.15°, 47.45°, 56.4°, 62.75°, 67.7°, 69.01°, 72.46°, 76.86° and 81.27° corresponds to (100), (002), (101), (102), (110), (103), (200), (112), (201), (004) and (202) planes of hexagonal of ZnO, respectively. The strongest peak at 2*θ* = 36.15° belongs to the (101) plane of the product.^62^ This diffraction was shifted to higher two theta values due to N doping on ZnONCBs and the formation of N-ZnONCBs catalyst.^63^ The diffraction spectrum also shows broadened peaks and this confirms the formation of the nano-sized N-ZnONCBs catalyst structure which has the wurtzite phase (JCPDS: 36-1451) and increased its crystallinity. The synthesized N-ZnONCBs catalyst diffraction peaks pattern is similar to that previously reported.^64^ Other diffraction peaks were observed due to the hydrazine hydrate peaks.^65^ The diffraction peaks were not altered and shifted the two theta values and the crystalline size of N-ZnONCBs catalyst was calculated from highest intensity diffraction peaks with the Scherer formula eqn (2).2
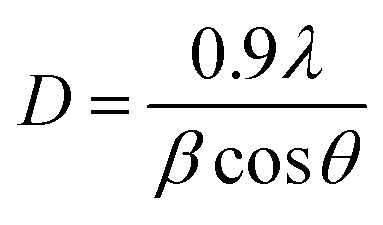
where *D* is the crystalline size of N-ZnONCBs catalyst, *λ* is the wavelength of the X-ray beam operating system, *β* is full-width half maximum calculated from highest intensity peak and *θ* is the angle of diffraction. For N-ZnONCBs catalyst the size was calculated as 61.6 nm. This result was in agreement with the TEM results. Additionally, the X-ray diffraction peaks of N-ZnONCBs catalyst sharpened with slight shifting and small intensity peaks were observed in comparison to the pure ZnONPs. This showed that the N-ZnONCBs catalyst decreased the size to 24 nm when compared to the pure ZnONPs which have a size of 30 nm as shown in Fig. 2(A and B).

### 3.5. Kubelka–Munk function

**Fig. 3:**
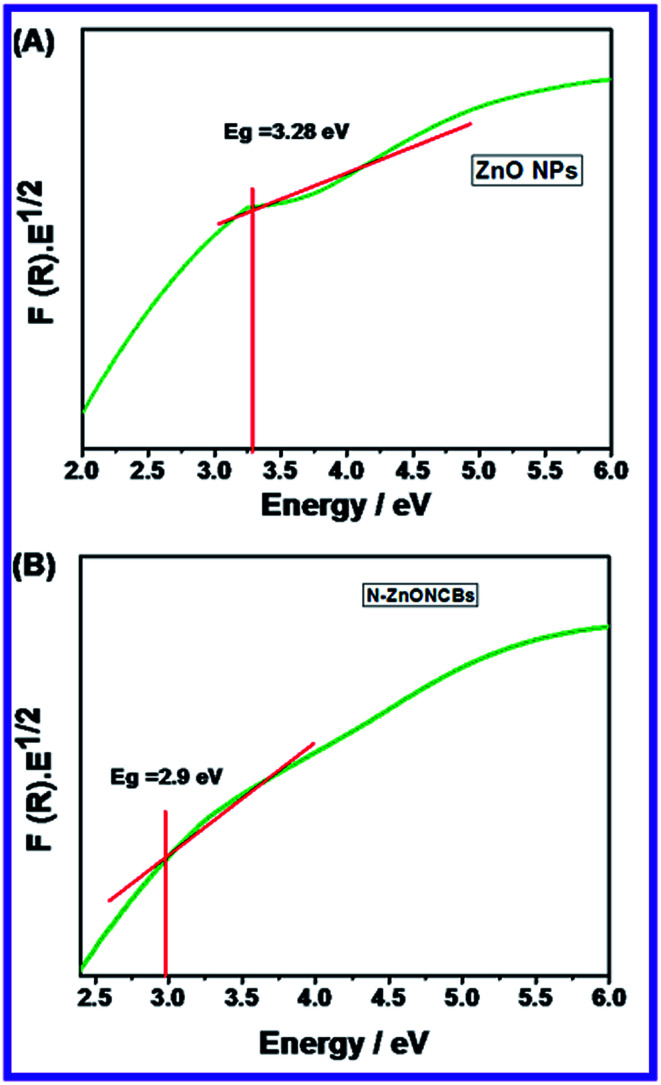
Tauc plot of the Kubelka–Munk function for (A) Pure ZnO and (B) N-ZnONCBs.

The Tauc plot of the Kubelka–Munk function was used to calculate the band gap energy before and after doping of nitrogen on ZnONCBs catalyst. According to the Kubelka–Munk function relation.3
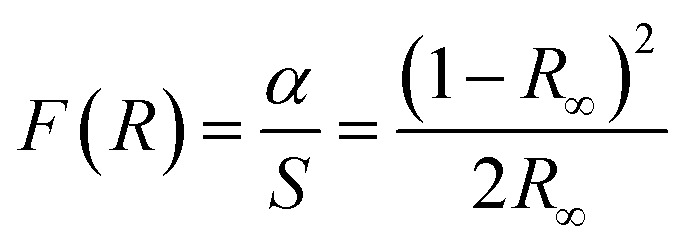
where *F*(*R*_∞_) is the Kubelka–Munk function and it was directly proportional to the absorption coefficient, and inversely proportional to the scattering coefficient (*S*), *R*_∞_ is the diffused reflectance of the pure ZnO and nitrogen doping on ZnONCBs. The band gap energy of pure ZnO is 3.28 eV and nitrogen-doped ZnONCBs catalyst is 2.9 eV, respectively as shown in Fig. 3(A and B).

### 3.6. Raman characterization

**Fig. 4:**
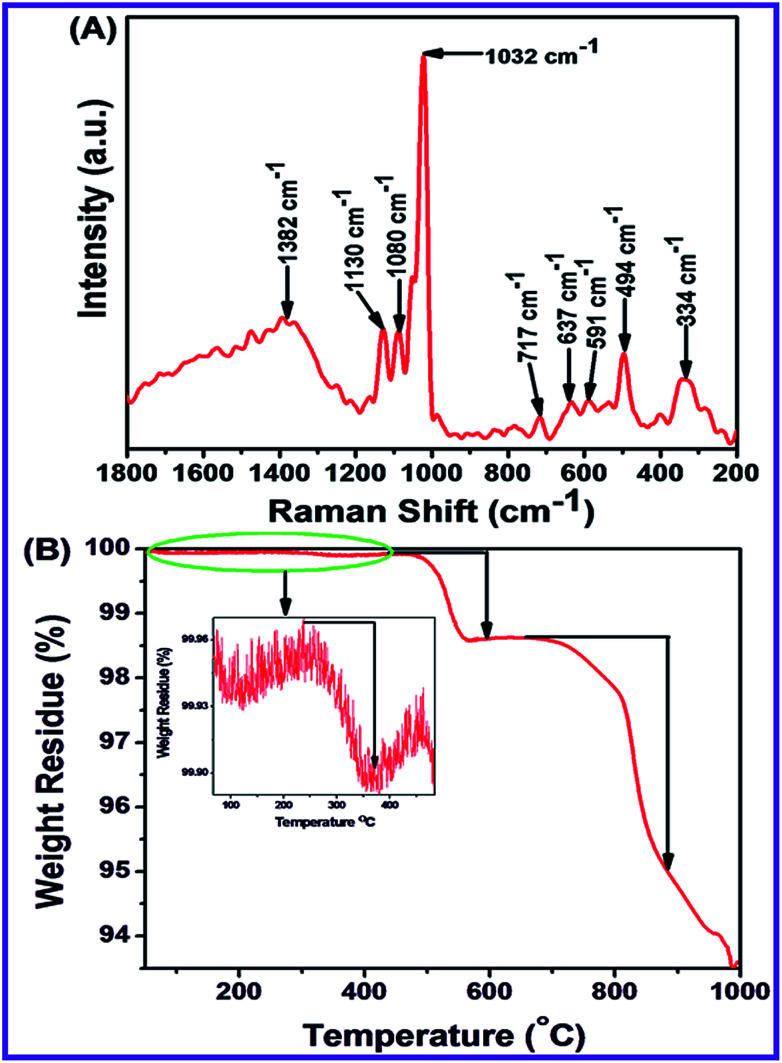
(A) Raman spectrum of N-ZnONCBs and (B) TGA spectrum of N-ZnONCBs.

Raman spectroscopy is an important approach to identify Raman active modes of polar and non-polar compounds. It also investigates the crystal symmetry, crystalline systems of microparticles and nanoparticles, defects and impurities. This spectroscopy was used to determine the vibration mode of N-ZnONCBs catalyst as shown in Fig. 4(A). N-ZnONCBs catalyst was based on the wurtzite shape and it has *C*^4^_6v_ space group (*P*6_3_*mc*). N-ZnONCBs catalyst was present with one primitive cell and two units, wherein N-ZnONCBs catalyst obeyed the *C*_3v_ symmetry. *Γ* irreducible representation of Brillouin zone of N-ZnONCBs catalyst existed the special vibration modes like A_1_ + 2B_1_ + E_1_ + E_2_, wherein A_1_ and E_1_ modes are vibrating in the infrared active and A_1_, E_1_ and E_2_ modes behaved as Raman active modes.^66^ Further, A_1_ and E_1_ modes split into longitudinal optical (LO) and transfers optical (TO).^67^ The Raman spectrum of N-ZnONCBs catalyst exhibited several peaks, wherein the wurtzite phase of N-ZnONCBs catalyst appeared as a less sharp peak at 494 cm^−1^ due to E_2H_ vibration mode of the Zn–O bond as shown in Fig. 4(A). Additionally, much less broadened peaks are observed at 334 cm^−1^ which might be attributed to E_2H_–E_2L_ mode in N-ZnONCBs catalyst of Zn–O band vibration.^68^ 591 cm^−1^ and 637 cm^−1^ peaks had been assigned to A_3_ and A_1_ (LO) vibration modes, respectively and this is due to the N doping on ZnONCBs catalyst.^69^ The Raman spectrum additionally exhibited a highly intense peak at 1032 cm^−1^ and a much less intense peak at 1080 cm^−1^ and these are assigned to the multiphonons of LO vibration of A_1_ and E_1_ modes of the Zn–O band.^70^ The asymmetric vibration mode of LO phonons gave two types of peaks at 1382 cm^−1^ and 1130 cm^−1^, which further confirmed the formation N-ZnONCBs catalyst.^71^

### 3.7. Thermogravimetric characterization

Thermal Gravimetric Analysis (TGA) is very essential to investigate the stability and weight loss of nanomaterials. N-ZnONCBs catalyst showed three kinds of weight loss curves as shown in Fig. 4(B). The very small weight loss (0.07%) in the range of 235 °C to 370 °C is due to the removal of water from the highly volatile surface of N-ZnONCBs catalyst in the temperature range of 50 °C to 1000 °C at inert atmosphere. The other weight loss (3.7%) observed in the temperature range of 450 °C to 595 °C was due to the dehydration Zn(OH)_2_ to ZnO.^72^ The huge weight loss (35.8%) in the temperature range of 650 °C to 884 °C was observed because of the removal of hydrazine groups in N-ZnONCBs catalyst.^73^ No further decomposition or weight loss was observed at temperatures above 884 °C and this showed the high-temperature stability of the N-ZnONCBs catalyst.

### 3.8. BET characterization

**Fig. 5:**
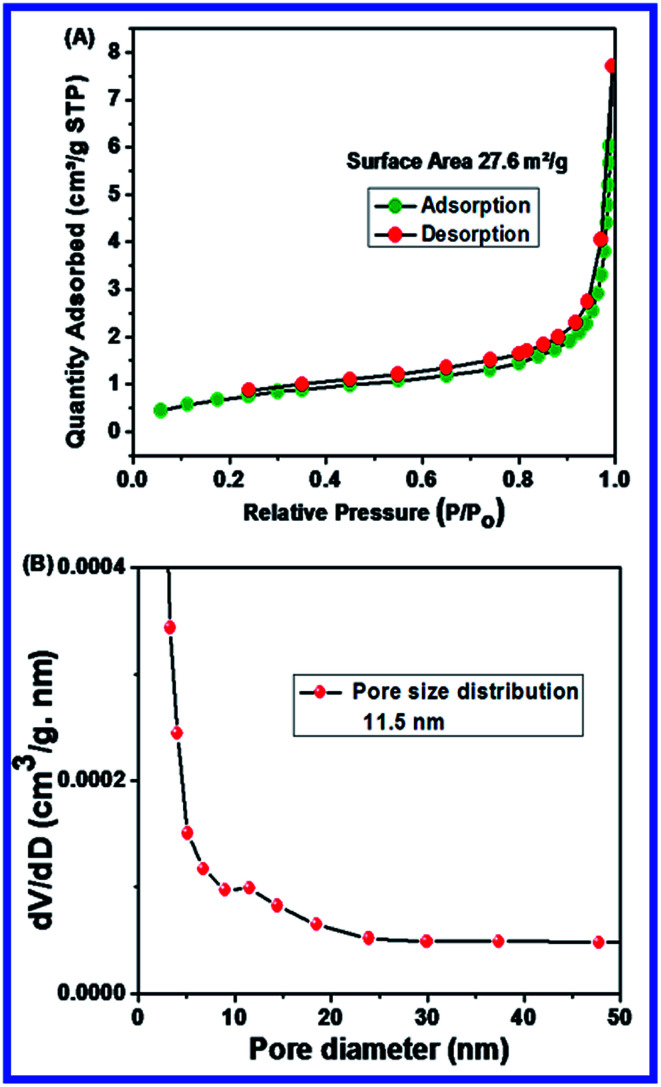
(A) Barrett–Joyner–Halenda (BJH) plot of surface area of N-ZnONCBs catalyst with N_2_ adsorption–desorption isotherms and (B) pore size distribution plots N-ZnONCBs catalyst.

The surface area and pore size of N-ZnONCBs catalyst had been investigated through the BET method under nitrogen gas sorption processes as shown in Fig. 5(A and B). The N-ZnONCBs catalyst surface area (27.6 m^2^ g^−1^) was obtained by using BJH distribution plots as shown in Fig. 5(A). The pore diameter plot clearly exhibited a small peak height of 11.5 nm as shown in Fig. 5(B). This characterization proved that the N-ZnONCBs catalyst had a porous nature which also accounts for the increased in the photocatalytic degradation of MB under visible light irradiation.

### 3.9. SEM characterization

**Fig. 6:**
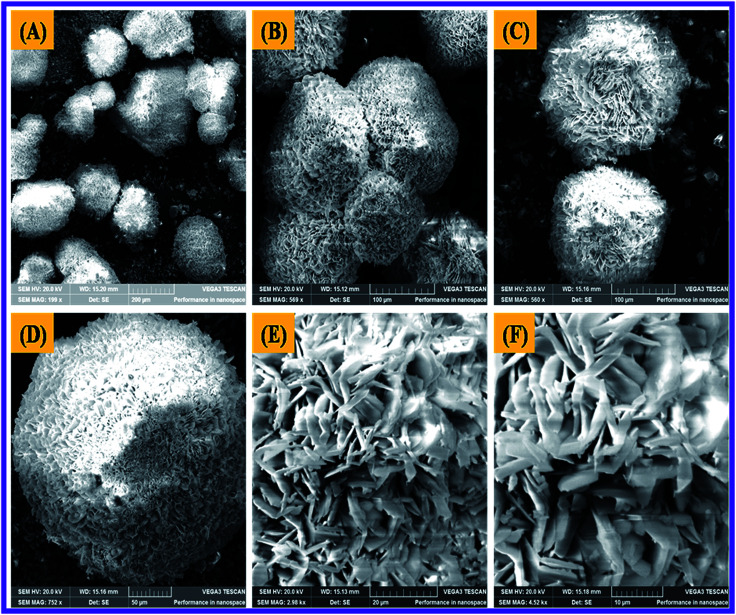
SEM images of N-ZnONCBs at different magnifications of (A) 200 μM, (B) 100 μM, (C and D) 50 μM, (E) 20 μM and (F) 10 μM.

**Fig. 7:**
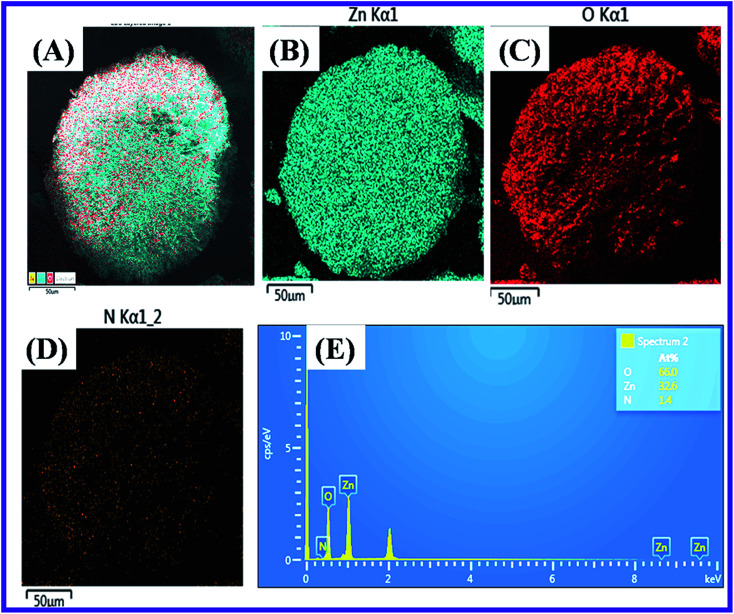
Elemental mapping images of (A) N-ZnONCBs at 50 μM magnification, (B) Zn, (C) O, (D) N and (E) EDX spectrum of N-ZnONCBs catalyst.

The Scanning Electron Microscope (SEM) characterization was an essential method to verify the morphology of the nanomaterials produced. The SEM image revealed the form of the N-ZnONCBs catalyst and it showed the cabbage structure as shown in Fig. 6(A–F). Several cabbage-like structures of N-ZnONCBs catalyst have been recorded at low magnification (200 μm) as shown in Fig. 6(A). The very compact four N-ZnONCBs catalyst cabbages structures have been demonstrated at higher magnification (100 μm) as shown in Fig. 6(B and C). Real cabbage images of N-ZnONCBs catalyst with good leaf formation was observed at higher magnification (50 μm) as shown in Fig. 6(D). The leaf of N-ZnONCBs catalyst was clearly observed at even higher magnification as depicted in Fig. 6(E and F). The N-ZnONCBs catalyst cabbage structure has been designed because of the nitrogen content in hydrazine hydrate as well as increase the time of hydrothermal reaction.^74,75^ The presence of the elements Zn, O and N in N-ZnONCBs catalyst was confirmed by elemental mapping and EDAX evaluation as shown in Fig. 7(A–E).

### 3.10. TEM characterization

**Fig. 8:**
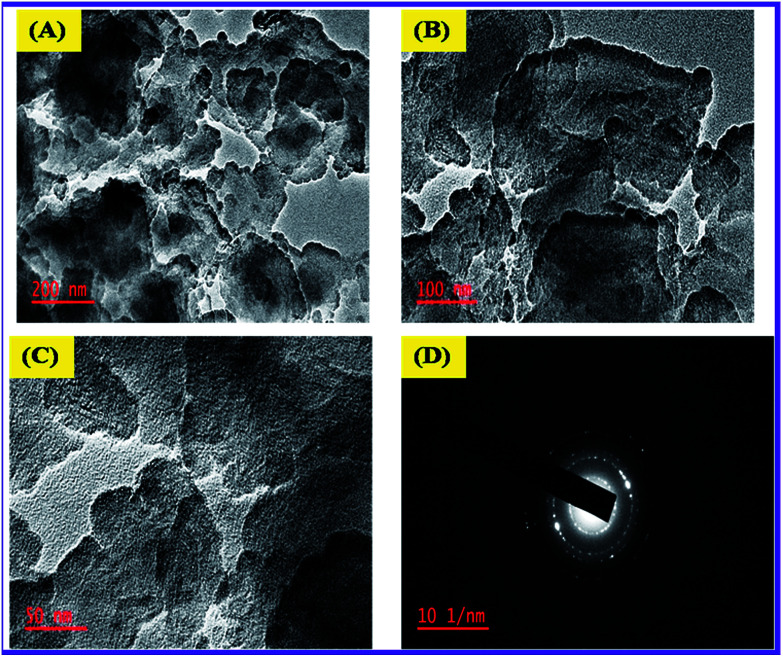
TEM images of N-ZnONCBs of (A) 200 nm, (B) 100 nm, (C) 50 nm and (D) SAED pattern of N-ZnONCBs.

Transmittance Electron Microscope (TEM) was used to determine the size of N-ZnONCBs catalyst as shown in Fig. 8(A–C). The N-ZnONCBs catalyst leaves changed into dispersed darkish dots at magnification (200 nm) as shown in Fig. 8(A). Fig. 8(B and C) have shown the clear cabbage leaves of N-ZnONCBs catalyst at higher magnification 100 nm and 50 nm. Those structures confirmed the formation of N-ZnONCBs catalyst nanocabbages. The dark shadow had been attributed to the nitrogen contents of ZnONCBs catalyst and provided additional evidence that N was present on ZnONCBs catalyst. Fig. 8(D) indicates the SAED pattern which showed the circle line with white spot particles and showed the well crystalline nature of N-ZnONCBs catalyst which is in agreement with the XRD results.

### 3.11. Photocatalytic applications

#### 3.11.1. Evaluation of the photocatalytic activity with N-ZnONCBs catalyst

**Fig. 9:**
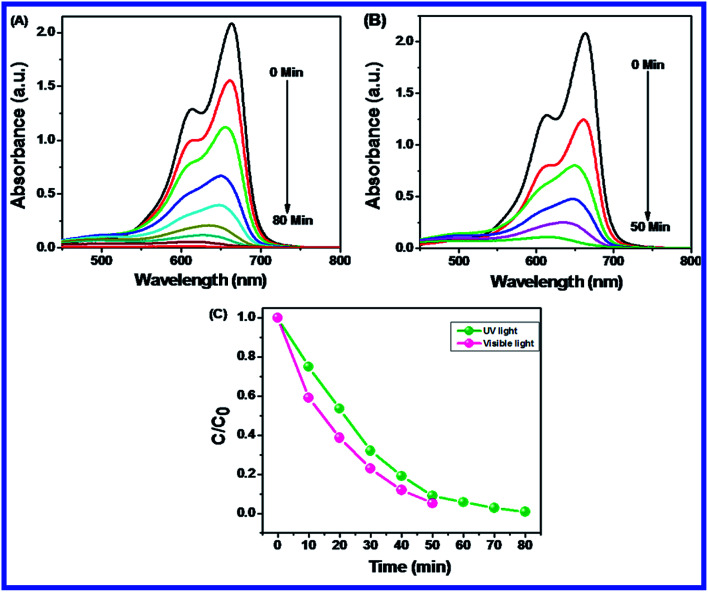
UV-visible spectra of MB with N-ZnONCBs catalyst (A) UV light at different time interval 0 to 80 minutes, (B) visible light with different time interval 0 to 50 minutes and (C) plot of *C*/*C*_0_*vs.* times photocatalytic degradation of MB.

The photocatalytic degradation of MB (15 ppm, 400 mL) was evaluated by using N-ZnONCBs (100 mg) catalyst with various light sources of UV and visible light irradiation as shown in Fig. 9(A and B). Fig. 9(A) shows the photocatalytic degradation of MB by N-ZnONCBs catalyst under UV light irradiation at various time periods 0 to 80 min. The photocatalytic degradation of MB was investigated with N-ZnONCBs catalyst under visible light irradiation with various time intervals 0 to 50 min. N-ZnONCBS catalyst was more suited for the photocatalytic degradation of MB under visible light irradiation within 50 minutes when compared to degradation under UV light irradiation as shown in Fig. 9(B). Similarly, the effectiveness of the photocatalyst was determined by using the plot of *C*/*C*_0_*vs.* time with N-ZnONCBs catalyst, where *C*_0_ is the initial concentration of MB and *C*_*t*_ is the final concentration of MB with based on degradation time *t*. N-ZnONCBs catalyst showed the higher photocatalytic activity under visible light irradiation within a short time interval 0 to 50 min when compared to UV light irradiation as shown in Fig. 9(C). The N-ZnONCBs catalyst therefore showed better photocatalytic behavior under visible light than the UV light irradiation.

#### 3.11.2. Effect of dosage of N-ZnONCBs catalyst for MB degradation

**Fig. 10:**
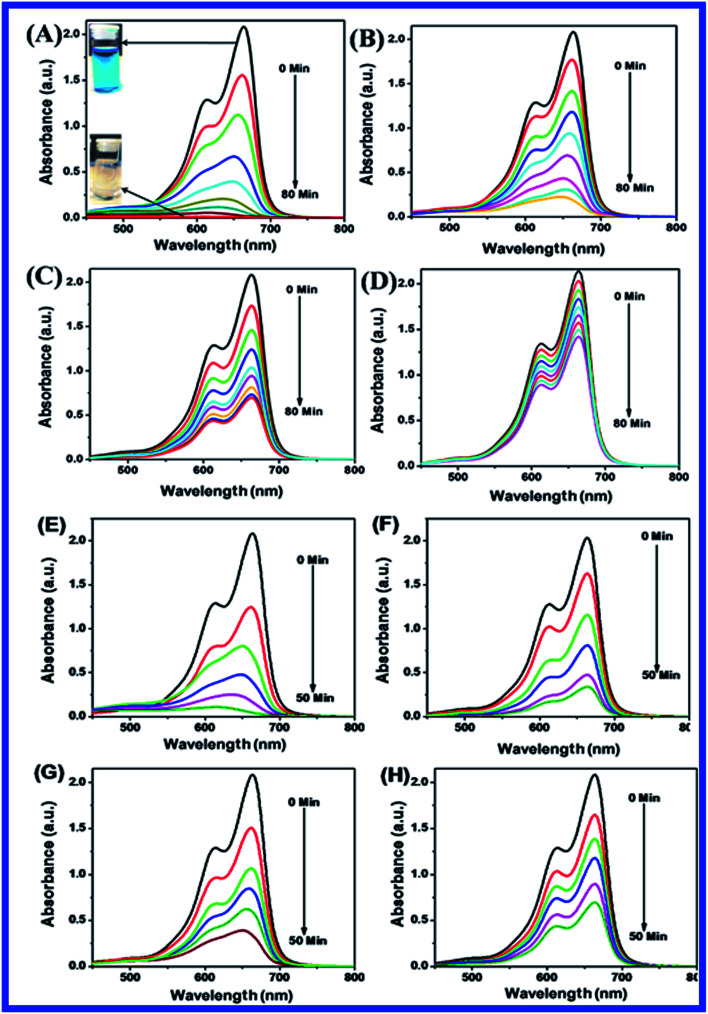
UV-visible spectra of MB with different catalyst of N-ZnONCBs (A) 100 mg, (B) 75 mg, (C) 50 mg and (D) 25 mg under UV light with reaction time interval 0 to 80 minutes and digital images of photocatalytic degradation of MB 0 min and 80 minutes with 100 mg (A) (inset). UV-visible spectra of MB for N-ZnONCBs with different catalyst dosage of (E) 100 mg, (F) 75 mg, (G) 50 mg and (H) 25 mg under visible light irradiation with different time interval 0 to 50 minutes.

Fig. 10(A–H) shows the photocatalytic degradation of MB using N-ZnONCBs catalyst and also evaluated the effect of dosages of N-ZnONCBs catalyst for photocatalytic degradation of MB under UV and visible light irradiation at different time intervals. Photocatalytic degradation of MB was achieved with different catalyst dosages of N-ZnONCBs catalyst under UV light irradiation 0 to 80 minutes as shown in Fig. 10(A–D). The N-ZnONCBs catalyst showed efficient photocatalytic abilities for the photocatalytic degradation MB under UV-irradiation for approximately 0 to 80 minutes. First, N-ZnONCBs catalyst was used to determine the photocatalytic degradation of MB under the dark conditions for 30 min under stirring without any UV light irradiation and it is labeled as the 0 minute peak in the UV-visible spectra as shown in Fig. 10(A). After this, the photocatalytic degradation of MB was continued at different times under UV-light irradiation and the corresponding UV-visible spectra have confirmed two peaks at 665 nm and 612 nm as depicted in Fig. 10(A). The two absorption peaks at 665 nm and 612 nm have been related to the monomer and dimer of MB.^76^ The two peaks of MB were reduced in intensity and the dye was completely decolorized with different times of UV-light irradiation which ranged from approximately 0 min to 80 min with digital photos of MB (0 min and 80 min) inset as shown in Fig. 10(A) (inset).

Fig. 10(B–D) shows the effect of dosage of N-ZnONCBs catalyst on the photocatalytic degradation of MB with the concentration of 15 ppm (400 mL) (3.75 mg L^−1^ at pH 7) and UV-irradiation time about 0 to 80 min in comparison with Fig. 10(A). Fig. 10(B) suggests that the photocatalytic degradation of MB was nearly completely degraded through the use of N-ZnONCBs catalyst with 75 mg (187.5 mg L^−1^) under the UV-light irradiation for about 0 to 80 minutes. UV-visible spectra of MB have displayed that the exclusive dosages of 50 mg (125 mg L^−1^) and 25 mg (62.5 mg L^−1^) under UV-light irradiation took approximately 0 to 80 minutes as shown in Fig. 10(C and D). As seen from (Fig. 10(C and D)), MB was not completely degraded within 80 minutes under UV light irradiation due to the insufficient dosage of N-ZnONCBs catalyst and this reduced the catalytic ability. Comparing the overall spectra of MB degradation (Fig. 10(A–D)), in which MB absorption peak at 665 nm regularly decreased in intensity within 80 min under UV irradiation due to 100 mg of N-ZnONCBs catalyst dosage which was enough for the degradation of MB dye as shown in Fig. 10(A).

Fig. 10(E–H) shows the photocatalytic degradation of MB which was investigated under visible light irradiation at different time interval 0 to 5 minutes. These results displayed higher and faster degradation of MB with N-ZnONCBs catalyst under the visible light irradiation when compared to UV irradiation as shown in Fig. 10(A–D). The N-ZnONCBs catalyst was therefore more efficient at degrading MB under visible light irradiation at time intervals of 0 to 50 minutes because the catalyst was more active under the visible light than under UV visible light irradiation. The photocatalytic degradation capacity of N-ZnONCBs catalyst was evaluated not only by using different dosages of the catalyst and but also by calculating the degradation of efficiency (%) of MB using the Beer–Lambert law.^77^

**Fig. 11:**
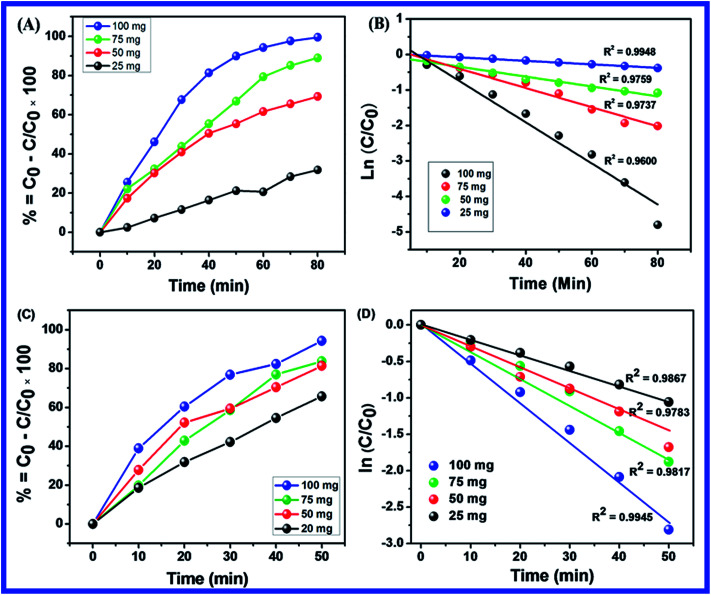
(A) Plot of percentage of degradation *vs.* times, (B) ln(*C*/*C*_0_) *vs.* times of MB with different N-ZnONCBs catalyst dosage 100 mg, 75 mg, 50 mg and 25 mg under the UV light irradiation, (C) plot of percentage of degradation *vs.* times and (D) ln(*C*/*C*_0_) *vs.* times of MB with different N-ZnONCBs catalyst dosage 100 mg, 75 mg, 50 mg and 25 mg under the visible light irradiation.

Different dosages of N-ZnONCBs catalyst (100 mg, 75 mg, 50 mg and 25 mg in 15 ppm of 400 mL) were used for the photocatalytic degradation of MB with UV light and visible light irradiation at the different times of 0 to 50 minutes as shown in Fig. 10(A–H). The highest dosage of N-ZnONCBs catalyst was 100 mg (98.6%) for the photocatalytic degradation of MB, when compared to 75 mg (89.08%), 50 mg (69.54%) and 25 mg (31.96%) under the UV light irradiation as shown in Fig. 11(A). The percentage of removal of MB 100 mg (96.22%), 75 mg (85.6%), 50 mg (81.93%) and 25 mg (68.4%) was calculated from the plot between efficiency (%) *vs.* time under visible light irradiation as shown in Fig. 11(B). The highest dosage of N-ZnONCBs catalyst resulted in almost complete photocatalytic degradation of MB due to increasing the active sites of the catalyst. In addition, the rate of decay of MB was investigated with different dosages of N-ZnONCBs catalyst under the UV light and visible light irradiation. The plot of ln(*C*/*C*_0_) *vs.* time was explored to calculate the pseudo-first-order rate constant for the degradation of MB as shown in Fig. 11(B and D). Langmuir–Hinshelwood version may be expressed by eqn (4).^78^

4 ln(*C*/*C*_0_) = −*kt*

where *C*_0_ is the initial concentration of the MB, *C* is the final concentration after UV and visible light irradiation and *k* is the rate constant. This plot demonstrated a higher photodegradation rate of MB with higher dosages of N-ZnONCBs (*k* = −0.0579 min^−1^ for 100 mg), when compared with other dosages (*k* = −0.0270 min^−1^ for 75 mg, *k* = −0.0139 min^−1^ for 50 mg and *k* = −0.0049 min^−1^ for 25 mg) under UV light irradiation as shown in Fig. 11(C). Fig. 11(D) shows the plot of ln(*C*_*t*_/*C*_0_) *vs.* time which demonstrated that the degradation rate constant of MB with corresponding to *k* = −0.0585 min^−1^ for 100 mg, *k* = −0.0384 min^−1^ for 75 mg, *k* = −0.032 min^−1^ for 50 mg and *k* = −0.026 min^−1^ for 25 mg respectively. N-ZnONCBs has therefore exhibited higher photocatalytic degradation activity under visible light irradiation. The excellent photodegradation efficiency and kinetic rate constant were displayed by using 100 mg of N-ZnONCBs when compared to that under the visible light and these results were compared with the previous report in Table 1.^79–89^

**Table 1:** Photocatalytic degradation of MB with different morphology of ZnO nanoparticles under UV light and visible light irradiation

Catalyst	Methods	% removal	Conditions	Degradation rate MB (*k* = min^−1^)	Ref.
ZnONPs^a^	Sol–gel method	95	UV light	0.12	79
ZnONPs	Precipitation method	92.5	UV light	0.0124	80
ZnONPs	Co-precipitate method	73.4	UV light	0.0159	81
ZnONSPs^b^	Polyol-method	99.1	UV light	0.07432	82
ZnONRDs^c^	Chemical method	80	UV light	0.01402	83
ZnONRDs	Hydrothermal method	93	Visible light	0.0208	84
ZnONFLs^d^	Biosynthesis method	83	UV light	0.00444	85
ZnONFLs	Precipitation method	98	UV light	—	86
ZnONPCl^e^	Green synthesis method	95	Sunlight	—	87
ZnOMFLs^f^	Thermal decomposition method	99	UV light	—	88
N-ZnONPs^g^	Mechanochemical method	98	Visible light	—	89
**N-ZnONCBs**	**Hydrothermal method**	**99.6**	**UV light and visible light**	**−0.0579 and −0.0585**	**This work**

a ZnO nanoparticles.

b ZnO nanospheres.

c ZnO nanorods.

d ZnO nanoflowers.

e ZnO nanopencils.

f ZnO microflowers.

g Nitrogen doped ZnO nanoparticles.

#### 3.11.3. Effect of pH of MB degradation

**Fig. 12:**
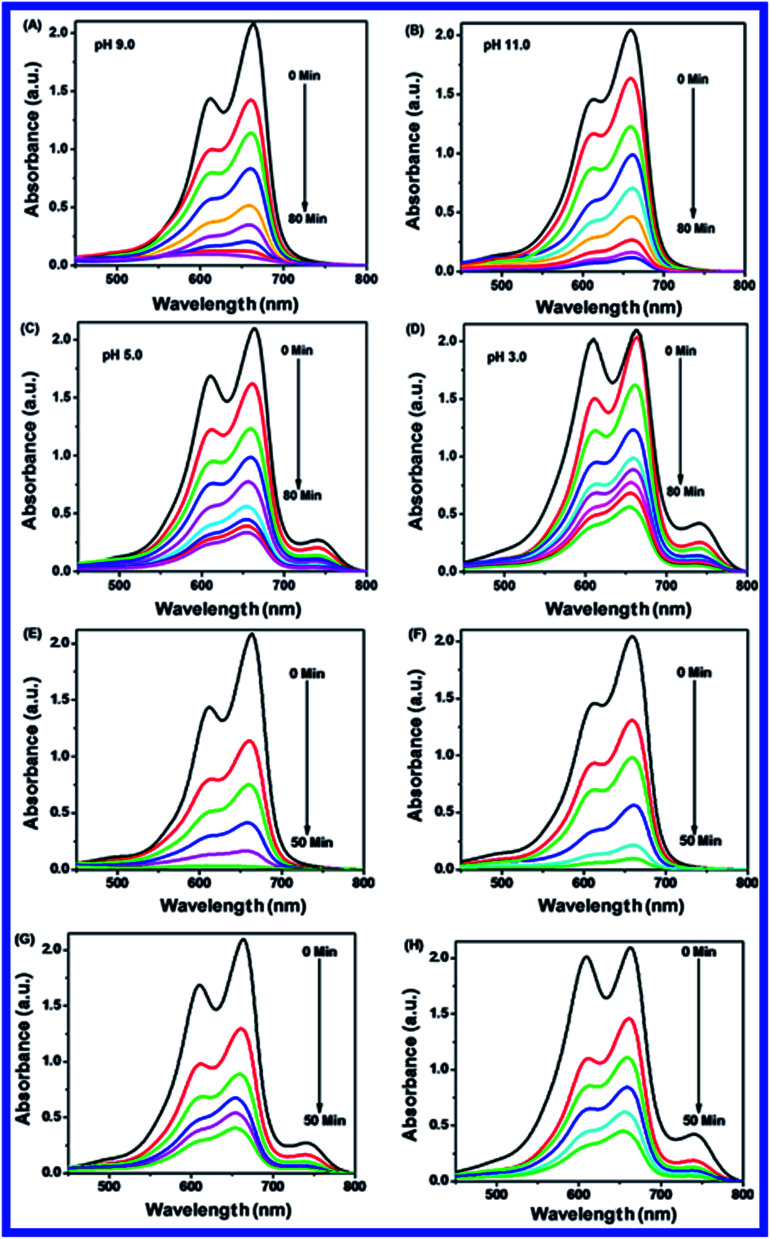
UV-visible spectra of MB with different pH of (A) 9, (B) 11, (C) 5 and (D) 3 with N-ZnONCBs under UV light irradiation at reaction time interval 0 to 80 minutes. UV-visible spectra of MB with different pH of (E) 9, (F) 11, (G) 5 and (H) 3 with N-ZnONCBs under visible light irradiation at reaction time interval 0 to 50 minutes.

**Fig. 13:**
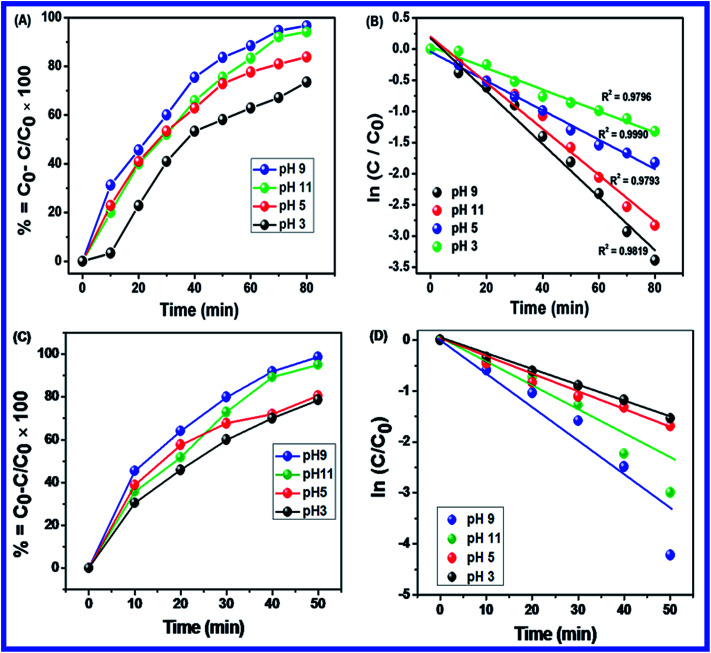
(A) Plot of percentage of degradation *vs.* times, (B) ln(*C*/*C*_0_) *vs.* times of MB with different pH of 9, 11, 5 and 3 under the UV light irradiation with reaction time interval 0 to 80 minutes, (C) plot of percentage of degradation *vs.* times and (D) ln(*C*/*C*_0_) *vs.* times of MB with pH of 9, 11, 5 and 3 under the visible light irradiation with reaction time interval 0 to 50 minutes.

Normally, the photocatalytic degradation was accomplished based on the pH of the solution and surface charge of the catalyst. This is important as the pH of wastewater varies. The effect of pH was studied for the photocatalytic degradation of MB solution with N-ZnONCBs catalyst. Consequently, the photocatalytic degradation of MB was displayed by using different pH solutions of 3, 5, 9 and 11 with 100 mg of N-ZnONCBs catalyst, 15 ppm of MB in 400 ml under UV and visible light irradiation with corresponding time intervals of 0 to 80 minutes and 0 to 50 minutes, respectively as shown in Fig. 12(A–H). At pH 5 and pH 3 MB was no longer completely degraded due to the positive charge of MB and the positive charge of N-ZnONCBs catalyst which repels the cationic dye as shown in Fig. 12(C and D). The photocatalytic degradation of MB at higher pH 9 and pH 11 of MB solution is shown in Fig. 12(A and B). At pH 9 and pH 11 it was proven that the degradation of MB with N-ZnONCBs catalyst under UV light and visible light irradiation took around 0 to 80 minutes and 0 to 50 minutes because of the negative charge of N-ZnONCBs catalyst at greater concentration of ^−^OH ions, which interact with the positive charge of MB for electrostatic attraction.^90^Fig. 12(G and H) shows the photocatalyst degradation which was explored with the N-ZnONCBs catalyst under visible light irradiation within 0 to 50 minutes. This showed better photocatalytic activity when compared to UV light irradiation at pH 5 and pH 3 as shown in Fig. 12(C and D). Fig. 12(E) shows great photocatalytic decolorisation of MB which was investigated with N-ZNONCBs catalyst under visible light irradiation at pH 9. This led to quick degradation of MB within 50 minutes when compared to the results in Fig. 12(C). At pH 9 a negative charge predominates on the surface of the N-ZnONCBs catalyst and it easily degraded the positively charged MB due to good electrostatic interactions between them. At pH 11 the N-ZnONCBs catalyst surface charge became negatively charged and positively charged MB became negatively charged to reduce the photocatalytic activity under the visible light irradiation as shown in Fig. 12(F). This degradation study exhibited greater photocatalytic ability when compared to the results in Fig. 12(B). With an increase in pH of up to 11 increase in the conduction band (e^−^) and valence band (h^+^) recombination as well as a decrease in photodegradation of MB was observed.^91^ As a result, the photocatalytic degradation of MB was optimized at pH 9 due to the negative charge of the N-ZnONCBs catalyst and the positive charge of MB which led to electrostatic attraction and easy degradation under the UV light and visible light irradiation as shown in Fig. 12(A and E). Fig. 13(A and B) shows the plots that were used to calculate the percentage removal of MB. These were calculated as 99%, 90%, 80% and 75% and kinetic rate constants were determined as *k* = −0.0425 min^−1^, *k* = −0.0370 min^−1^, *k* = −0.025 min^−1^ and *k* = −0.017 min^−1^ with pH 9, pH 11, pH 5 and pH 3 under UV light irradiation. Fig. 13(C and D) shows the plots that were used to calculate the percentage removal of MB which were calculated as 99%, 90%, 85% and 80% and kinetic rate constants were determined as *k* = −0.0599 min^−1^ (*R*^2^ = 0.9519), *k* = −0.0781 min^−1^ (*R*^2^ = 0.9078), *k* = −0.0323 min^−1^ (*R*^2^ = 0.9828), and *k* = −0.029 min^−1^ (*R*^2^ = 0.9977) with pH 9, pH 11, pH 5 and pH 3 under visible light irradiation. From these studies, the greatest MB degradation efficiency was observed with N-ZnONCBs catalyst under the visible light irradiation at the optimized pH of 9.

#### 3.11.4. Effect of concentration of MB degradation

**Fig. 14:**
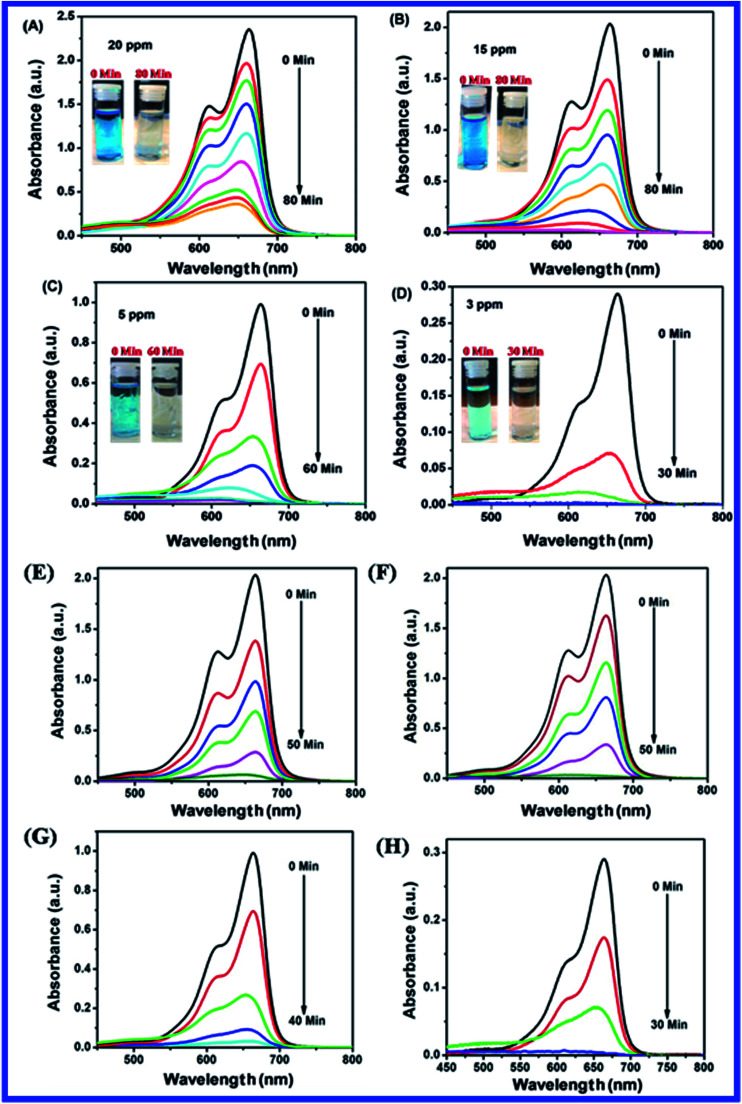
UV-visible spectra of MB with different initial concentration of (A) 15 ppm, (B) 20 ppm, (C) 10 ppm and (D) 5 ppm under the UV light irradiation with reaction time interval 0 to 80 minutes and digital images of photocatalytic degradation of MB 0 min and 80 minutes (A–D) (inset). UV-visible spectra of MB with different initial concentration of (E) 15 ppm, (F) 20 ppm, (G) 10 ppm and (H) 5 ppm under visible light irradiation with reaction time interval 0 to 80 minutes.

The effect of different initial concentrations of MB was tested for the photocatalytic activity with N-ZnONCBs catalyst under the UV light and visible light irradiation at different time intervals as shown in Fig. 14(A–H). Fig. 14(B and F) shows the photocatalytic degradation of 15 ppm of MB which was investigated with N-ZnONCBs catalyst under UV light and visible light irradiation at various times intervals, in which visible light irradiation resulted in the faster degradation of MB within 0 to 50 minutes when compared to UV light irradiation 0 to 80 minutes. The concentrations 5 ppm and 3 ppm were quickly degraded with N-ZnONCBs catalyst under UV light and visible light irradiation at various time intervals as shown in Fig. 14(C, D, G and H) because of lower concentrations of MB. As seen from the spectra in (Fig. 14 (G and H)) better performance for photocatalytic activity was under visible light irradiation when compared to the results in Fig. 14(C and D). The high concentrations of 20 ppm were used to evaluate the photocatalytic ability with N-ZnONCBs catalyst under UV light and visible light irradiation at different time intervals as shown in Fig. 14(A and E). In this study, the best photocatalytic degradation of MB was observed with N-ZnONCBs catalyst under the visible light irradiation when compared to that under UV light irradiation as shown in Fig. 14(E). The higher concentration of MB dye blocks the UV and visible light irradiation, as result of less light energy falling on N-ZnONCBs catalyst.^92^

**Fig. 15:**
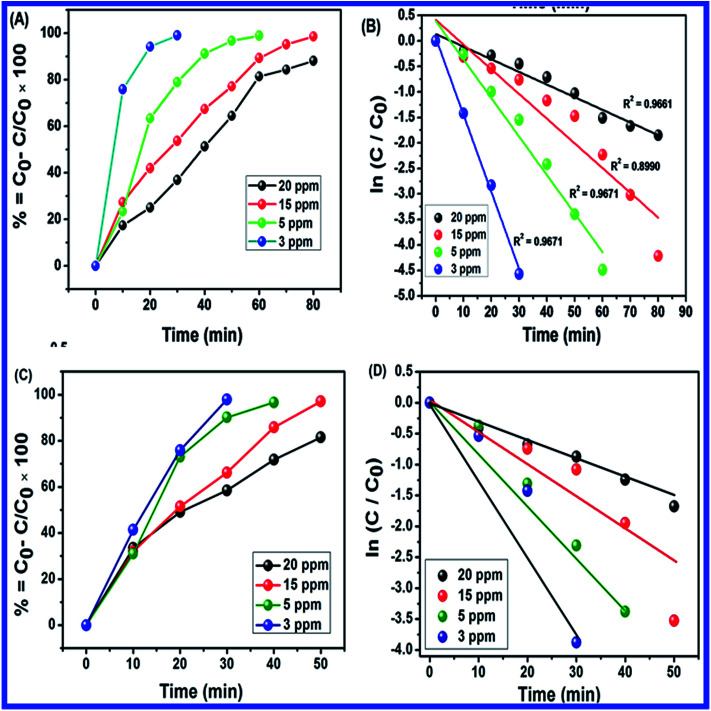
(A) Plot of percentage of degradation *vs.* times, (B) ln(*C*/*C*_0_) *vs.* times of MB with different concentration of 20 ppm, 15 ppm, 5 ppm and 3 ppm under the UV light irradiation with reaction time interval 0 to 80 minutes, (C) plot of percentage of degradation *vs.* times and (D) ln(*C*/*C*_0_) *vs.* times of MB with different concentration of 20 ppm, 15 ppm, 5 ppm and 3 ppm under the visible light irradiation with reaction time interval 0 to 50 minutes.

Fig. 15(A–D) shows the plots of percentage of removal *vs.* time of MB photocatalytic degradation with N-ZnONCBs catalyst under UV light and visible light irradiation. The percentage of removal of MB was 90%, 80%, 75% and 70% with corresponding concentrations of 20 ppm, 15 ppm, 5 ppm and 3 ppm using N-ZnONCBs catalyst under UV light irradiation at 0 to 80 minutes as shown in Fig. 15(A). The photocatalytic degradation percentage of removal was 98.5%, 99%, 98%, and 82% with corresponding to concentrations of 20 ppm, 15 ppm, 5 ppm and 3 ppm with N-ZnONCBs catalyst under the visible light irradiation 0 to 50 minutes as show in Fig. 15(B). The first order reaction was confirmed by linear plots of ln(*C*_0_/*C*) *vs.* time, from which were calculated the kinetic rate constant under UV light and visible light irradiation as shown in Fig. 15(C and D). The kinetic rate constant of *k* = −0.0248 min^−1^, *k* = −0.0485 min^−1^, *k* = −0.0756 min^−1^ and *k* = −0.1512 min^−1^ corresponding to concentrations of 20 ppm, 15 ppm, 5 ppm and 3 ppm were calculated for the degradation with N-ZnONCBs catalyst under UV light irradiation as shown in Fig. 15(C). The photocatalytic degradation rate constant of *k* = −0.0317 min^−1^ (*R*^2^ = 0.9838), *k* = −0.0647 min^−1^ (*R*^2^ = 0.8598), *k* = −0.0870 min^−1^ (*R*^2^ = 0.8703) and *k* = −0.1252 min^−1^ (*R*^2^ = 0.83118) corresponding to concentrations of 20 ppm, 15 ppm, 5 ppm and 3 ppm were calculated for degradation with N-ZnONCBs under visible light irradiation at 0 to 50 minutes as shown in Fig. 15(D). From the data, the percentage of removal and kinetic rate constant of MB were better when degradation was performed by N-ZnONCBs catalyst under visible light irradiation. The observations of (Fig. 15(A and B)) simply indicated that the optimized initial concentration of MB solution is (15 ppm).

#### 3.11.5. Stability and reusability of studies of N-ZnONCBs catalyst

**Fig. 16:**
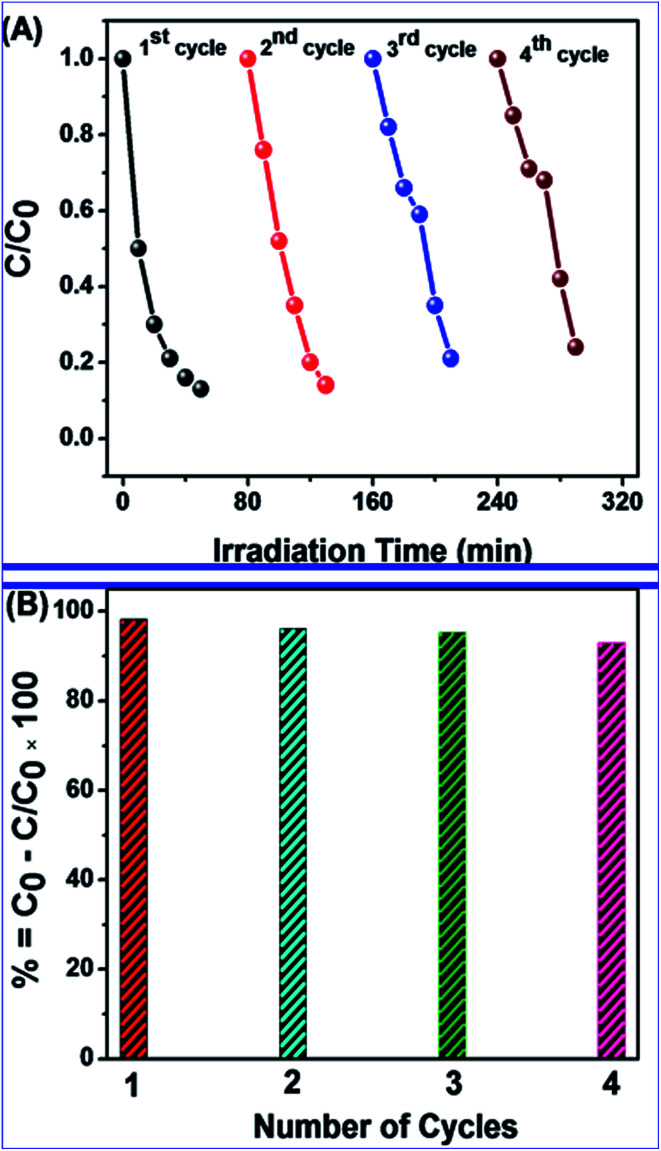
N-ZnONCBs catalyst reusability (A) repeat cycle experiment of MB degradation rate *vs.* irradiation times and (B) percentage of degradation rate *vs.* cycle numbers under visible light irradiation 0 to 50 minutes.

Repeat photocatalytic degradation of MB was investigated for determining the stability and reusability of N-ZnONCBs catalyst under the above conditions. The N-ZnONCBs catalyst was centrifuged by using water obtained at different times of purification, after that it was brought into contact with another fresh MB solution. This approach was followed at each cycle of photocatalytic degradation. Here, four repeat cycles of photodegradation of MB were completed under UV light irradiation within 80 minutes. The photocatalytic degradation kinetic rate of MB was obtained from the plots percent degradation rates *vs.* number of cycles and concentration *vs.* irradiation times as shown in Fig. 16(A and B). The degradation efficiency (93.2%) did not decrease much after 4 cycles of experiments as shown in Fig. 16(B). This observation confirmed that the N-ZnONCBs catalyst had excellent photostability, reusability and good efficiency for degradation of MB.

#### 3.11.6. Fluorescence quenching studies

**Fig. 17:**
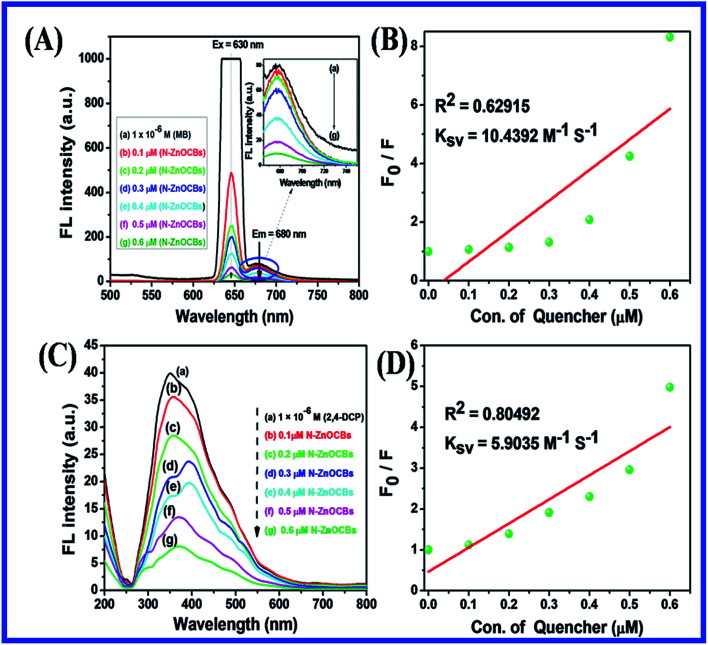
Fluorescence quenching spectra of MB (A) different concentration of N-ZnONCBs in water; (a) 1 × 10^−6^ M of MB, (b) 0.1 μM, (c) 0.2 μM, (d) 0.3 μM, (e) 0.4 μM, (f) 0.5 μM and (g) 0.6 μM at excited state at 630 nm, (B) Stern–Volmer plot of *F*_0_/*F vs.* concentration of N-ZNONCBs, (C) fluorescence spectra of 2,4-DCP and (D) Stern–Volmer plot of *F*_0_/*F vs.* time of various concentration of (a) 1 × 10^−6^ M of 2,4-DCP, (b) and (a) 1 × 10^−6^ M of MB, (b) 0.1 μM, (c) 0.2 μM, (d) 0.3 μM, (e) 0.4 μM, (f) 0.5 μM and (g) 0.6 μM at excited state at 240 nm.

The fluorescence quenching spectra of MB and 2,4-dichlorophenol (1 × 10^−6^ mol L^−1^) with different concentrations of N-ZnONCBs catalyst (0.1 μM to 0.6 μM) were obtained at excitation wavelengths 650 nm and 320 nm as shown in Fig. 17(A and B). The fluorescence quenching of study of MB with different concentration of N-ZnONCBs catalyst quencher at excitation wavelength 630 nm and emission wavelength 710 nm as shown in Fig. 17(A). The fluorescence intensity was decreased with increasing concentration of quencher concentration of N-ZnONCBs catalyst due to the quenching effect between them with cross relaxation and non-radiative transition processes as shown in Fig. 17(A). Fig. 17(B) shows the fluorescence quenching of 2,4-DCP was successively tested with different concentrations of N-ZnONCBs catalyst (1 to 6 μM). The fluorescence intensity of 2,4-DCP was decreased with increasing concentration of quencher N-ZnONCBs because of weak acidic dissociation pKa of 2,4-DCP to form 2,4-dichlorophenolate ion from 2,4-dichloropheneol at excitation wavelength 240 nm and emission wavelength 350 nm as shown in Fig. 17(B). However, 2,4 dichlorophenolate ion was easily attached to N-ZnONCBs quencher and reduced the fluorescence intensity. The fluorescence quenching activity of MB and 2,4-DCP with N-ZnONCBs catalyst were calculated by using Stern–Volmer equation.5
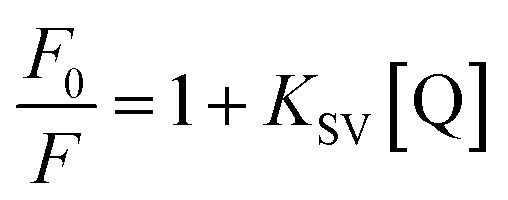
where, *F*_0_ and *F* are the fluorescence peak intensities of MB and 2,4-DCP in the absence and presence of quencher of N-ZNOCBs catalyst, *K*_SV_ is the Stern–Volmer constant or quenching rate constant, [Q] is the concentration of quencher. The linear plots of *F*_0_/*F vs.* Q gave Quenching rate constant, *K*_SV_ = 10.43 92 × 10^−6^ M^−1^ s^−1^ and *K*_SV_ = 5.9035 × 10^−6^ M^−1^ s^−1^ which corresponded to MB and 2,4-DCP respectively. These were obtained from the slope of the plots as shown in Fig. 17(C and D). From the plots of fluorescence it was noted that the quenching followed a dynamic quenching mechanism and reduced the electron–hole recombination rate.

#### 3.11.7. Photocatalytic degradation intermediate products of MB analysis by using LC-MS

**Fig. 18:**
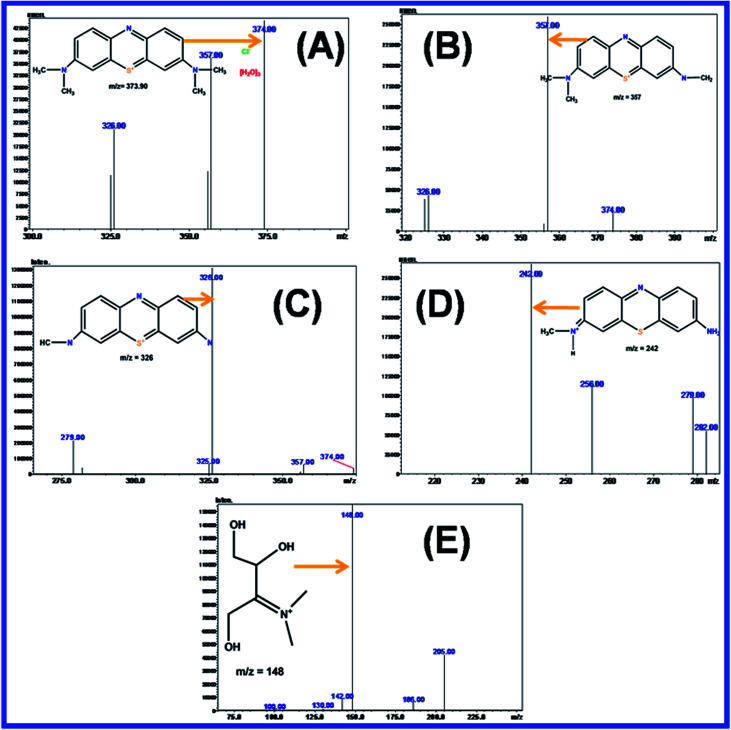
LC-MS spectra MB (A) 10 min, (B) 20 min, (C) 30 min, (D) 40 min, (E) 50 min under the visible light irradiation N-ZnONCBs catalyst = 150 mg/400 mL, MB = 10 ppm, different time 0 to 50 min and pH 7.

Liquid chromatography-mass spectrometry (LC-MS) is an important analytical technique for the identification of individual compounds with mass and identification of structures from mixtures and multiple compounds. It can also used to investigate organic, inorganic, biological and environmental compounds. The photocatalytic degradation of MB intermediate products was investigated by LC-MS analysis with N-ZnONCBs catalyst under visible light irradiation at different times of 0 to 50 minutes as shown in Fig. 18(A–E). Fig. 18(A) shows that the highest intensity peak was observed for the photocatalytic degradation of MB under the 10 minute visible light irradiation with mass (*m*/*z* = 374) and also different mass peaks were dependent on the composition and concentration of intermediate products. Fig. 18(B–E) shows the different intermediate products peaks which were recorded with different visible light irradiation times of 20 min, 30 min, 40 and 50 min respectively. After 50 minutes of visible light irradiation of MB dye was completely degraded into CO_2_ and H_2_O with N-ZnONCBs catalyst as shown in Fig. 18(E). For reason that MB dye is a color pollutant because of chromophoric and auxochrome groups of N and S bonds which are contained in lone pair electron aromatic benzene rings.^93^ After the photocatalytic degradation of MB was completely decolorized with N-ZnOCBs catalyst under visible light irradiation, identification of intermediates were also determined. During the photocatalytic degradation of MB the ring opens as the heterocyclic aromatic group, which was formed by electron transfer conversion of C–S^+^ = C to C–(S=O)–C The oxidizing agent of hydroxy radicals (˙OH) effectively changed the oxidation state of the sulfur atom (2 to 0), which induced the opening of the heterocyclic aromatic groups of S and N atoms. The H atom was interacted with carbon and nitrogen to give C–H and N–H, in which the hydrogen atom was generated by the reduction of the water molecule. These types of cleavage occurred during the photocatalytic degradation of MB, when the number of intermediate products of mass number were (*m*/*z* = 374), (*m*/*z* = 356), (*m*/*z* = 326), (*m*/*z* = 242), (*m*/*z* = 148) and (*m*/*z* = 130) as shown in Fig. 18(A–E). Therefore, MB was completely mineralized in the photocatalytic degradation process.^94^

#### 3.11.8. Complete mineralization with TOC and tested with colorless pollutant of 2,4-DCP

**Fig. 19:**
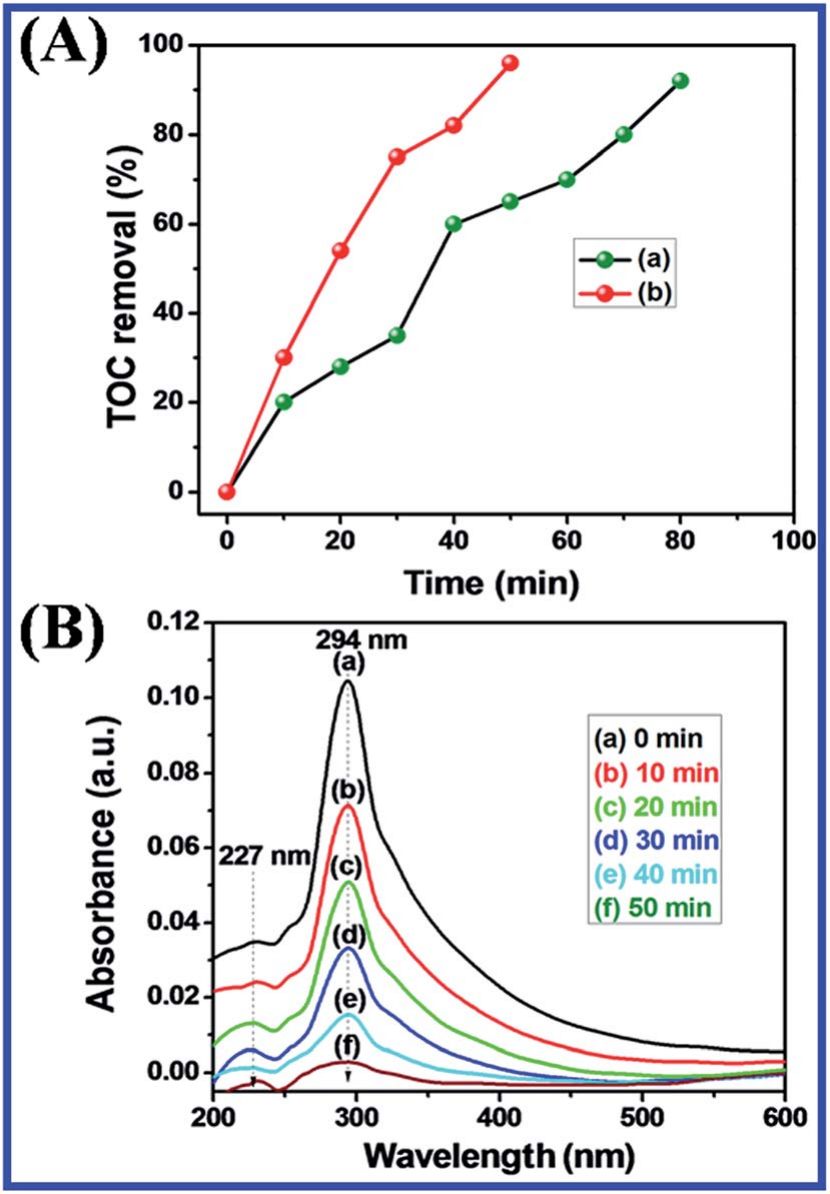
(A) Percentage of TOC removal of MB with N-ZNOCBs catalyst under UV light irradiation (a) and visible light irradiation (b) and (B) UV-visible spectra of 1 × 10 × M of 2,4-DCP with 100 mg of N-ZnONCBs under visible light irradiation at 0 to 50 minutes.

Fig. 19A(a and b) shows that the complete photocatalytic degradation of MB was further confirmed by a TOC analyzer with N-ZnONCBs catalyst to determine the complete mineralization of MB dye under UV light and visible light irradiation at different time intervals. The percentage of removal of carbon contents (92.5%) MB was obtained by TOC removal under UV light irradiation at different time intervals of 0 to 80 minutes as shown in Fig. 19A(a). The percentage removal of total organic carbon (96.2%) of MB was obtained from the TOC analyzer under the visible light irradiation at various time intervals 0 to 50 min as shown in Fig. 19A(b). The results showed again that the visible light irradiation of MB exhibited the higher removals of TOC when compared to UV light irradiation. Hence, N-ZnONCBs catalyst gave highest photocatalytic activity under visible light irradiation. The photocatalytic degradation of a colorless pollutant of 2,4-DCP was tested with N-ZnONCBs catalyst under the visible light irradiation at 0 to 50 minutes as shown in Fig. 19B. The intensity peaks of 2 nm, 265 nm of 2,4-DCP was decreased with increased times due to intermediate products of dichloride benzoquinone, CO_2_, and H_2_O.

#### 3.11.9. Zeta-sizer analyzer

**Fig. 20:**
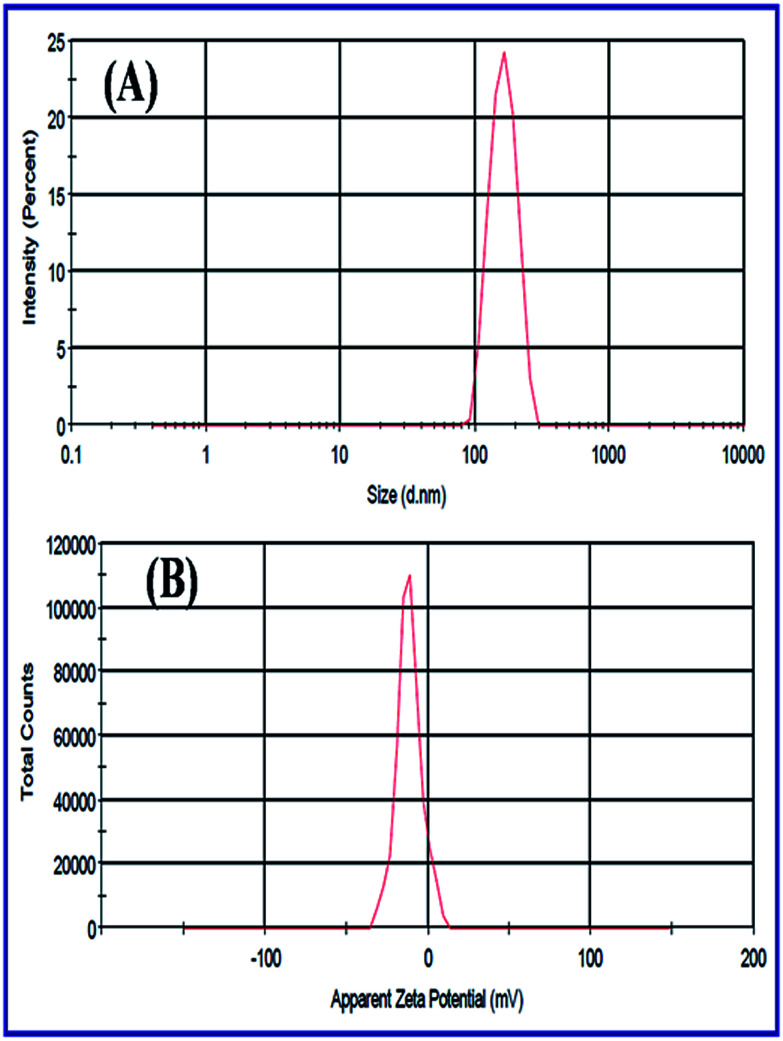
(A) DLS spectrum of N-ZnONCBs and (B) zeta-potential of N-ZnONCBs dispersed in 10 ml (50 mg) of water medium with sonication 10 minutes.

The Zeta-sizer was used to determine the average size diameter of nanoparticles with Dynamic Light Scattering (DLS) approach and also investigated the zeta potential with electrophoretic mobility by using Smoluchowski equation. Fig. 20(A and B) shows the DLS and zeta potential which were determined by the average size distribution and charge of N-ZnONCBs catalyst dispersed in 10 ml (50 mg) of water medium with sonication 10 minute before zeta analyzing. The average size distribution of the N-ZnONCBs catalyst was determined by dynamic light scattering (DLS) with average size 384 nm as shown in Fig. 20(A). The zeta potential of N-ZnONCBs catalyst was −11.5 mV a shown in Fig. 18(B). This confirmed the N-ZnONCBs catalyst negative charge to increase the repulsion between the particles and better attraction with the positive charge of MB dye for enhancing photocatalytic degradation.

**Fig. 21:**
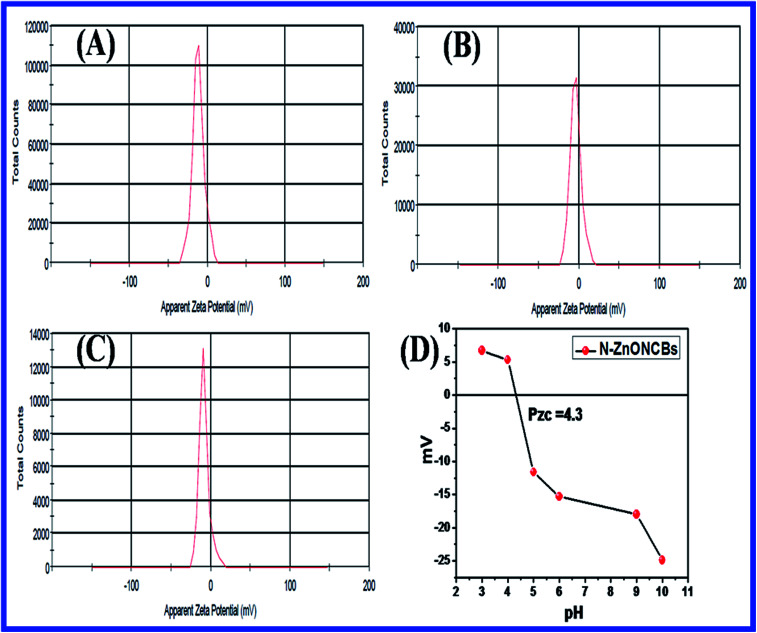
(A) Zeta potential of N-ZnONCBs, (B) N-ZnONCBs-MB solution initial stage, (C) N-ZnONBCs-MB solution after 30 minutes adsorption and (D) to determine the zero point charge with different pH 3, pH 4, pH 5, pH 6, pH 9 and pH 10 of N-ZnONCBs with various zeta-potential.

Fig. 21(A–C) indicates the zeta potentials which had been evaluated with pure N-ZnONCBs catalyst, initially adsorption of MB dye and after adsorption of MB with 30 minutes at pH 7.0. The N-ZnONCBs catalyst exhibited the negative potential (−11.5) mV due to the negative charge on the surface of N-ZnOnBCs catalyst as shown in Fig. 21C(a). The MB solution was mixed with N-ZnONCBs catalyst and slightly change zeta potential (−3.94 mV) because of positive charge of the Sulfur atom of MB adsorbed on N-ZnONCBs catalyst surface as shown in Fig. 21C(b). The MB solution was adsorbed on N-ZnONCBs catalyst after 30 minutes to increase the negative potential (−8.39 mV). The nitrogen atom of N-ZnONCBs catalyst interacted better with the sulfur atom of the color of MB solution leading to the formation of MBH colorless solution.^95^Fig. 21D shows the point of zero charge (Pzc) of N-ZnONCBs catalyst which was measured at varying pH (4.5). This was evaluated as 4.3 clearly showing that the surface of N-ZnONCBs catalyst was positively charged below pH 4.3 thereby rendering it unsuitable for electrostatic interactions with cationic MB. At higher pH N-ZnOCBs catalyst becomes more negatively charged and it was easily attracted to the positive charge of MB dye to enhance photocatalytic activity.

#### 3.11.10. Photocatalytic degradation mechanism of MB

The mechanism of the photocatalytic degradation of MB with N-ZnONCBs catalyst under the UV light and visible light irradiation is shown in Fig. 24. The UV light irradiation energy has the highest energy to reduce the band gap of N-ZnONCBs catalyst (3.29 eV) and this easily absorbed the UV light energy to create the valence band (h^+^) and conduction band (e^−^). The conduction band e^−^ interacts with dissolved oxygen to form superoxide radicals (O^2−^˙) and the valence band hole (h^+^) can oxidize the water molecule to produce the hydroxide radical (˙OH).^96^ Both radicals of (O^2−^˙) and (˙OH) were shown to be good oxidizing agents that readily react with MB molecule to produce the degradation products of CO_2_, H_2_O, Cl, SO_4_^2−^ and NO^3−^. The photocatalytic degradation of MB reaction steps are shown in Fig. 20.

**Fig. 22:**
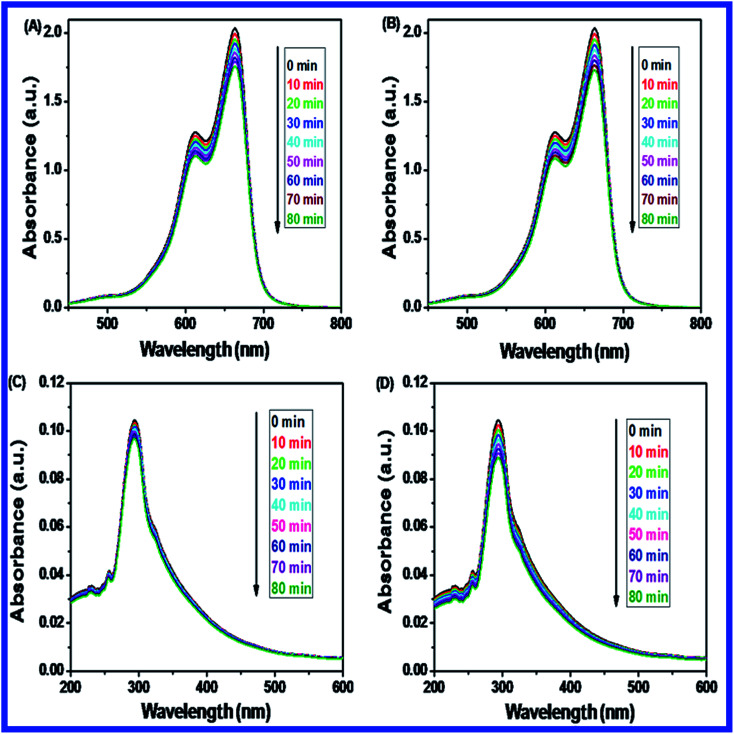
Comparison for the photolysis of MB and 2,4-DCP under UV and visible light irradiation at 0 to 80 min; (A) photolysis of MB under UV light, (B) photolysis of MB under visible light, (C) photolysis of 2,4-DCP under UV light, (D) photolysis of 2,4-DCP under visible light without N-ZnONCBs catalyst.

#### 3.11.11. Photolysis of MB and 2,4-DCP under UV light and visible light irradiation

**Fig. 23:**
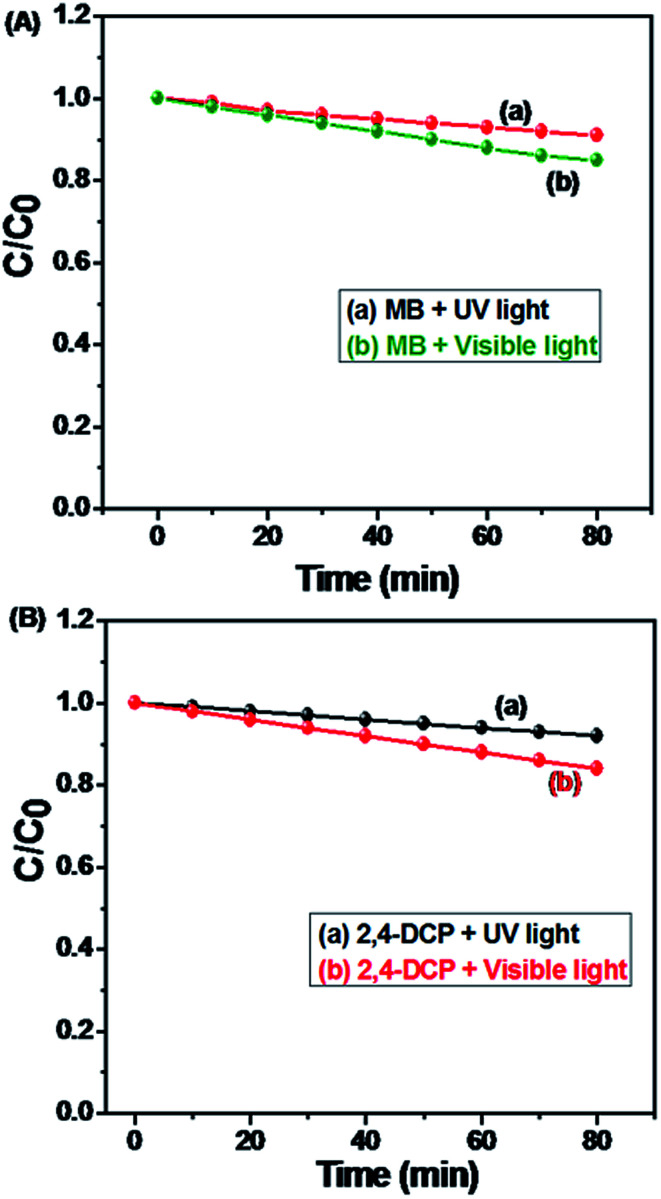
Calibration plot of *C*/*C*_0_ photolysis of MB and 2,4-DCP under the UV and visible light irradiation with different time interval 0 to 80 minutes.

**Fig. 24:**
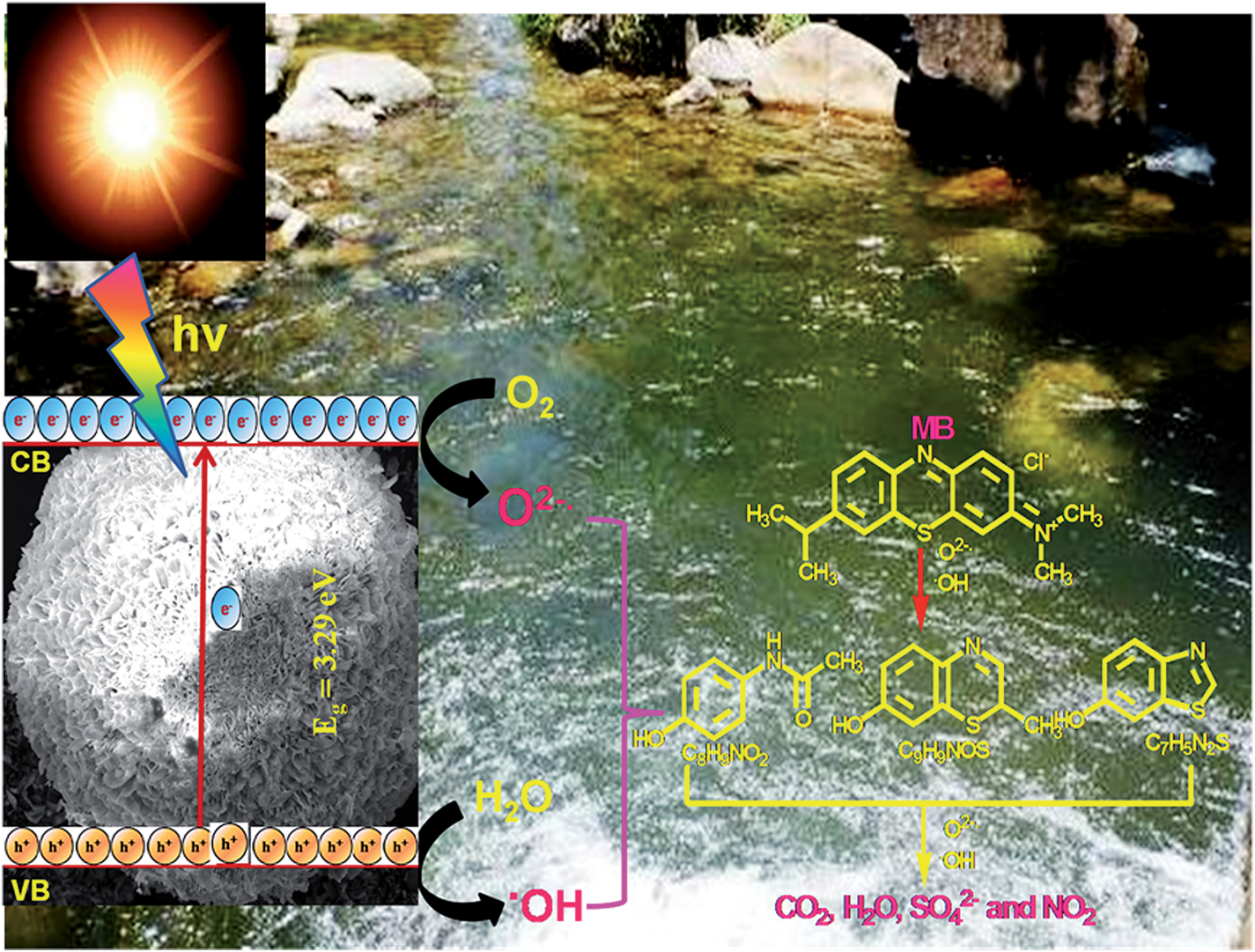
Mechanism of photocatalytic degradation of MB using N-ZnONCBs catalyst under visible light irradiation.

Photolysis of MB and 2,4-DCP had been performed under UV and visible light irradiation with different times ranging from 0 to 80 minutes without N-ZnONCBs catalyst as shown in Fig. 22(A–D). Fig. 22(A) indicates that the photolysis response of MB was carried out without a catalyst under UV light irradiation at various times intervals of 0 to 80 minutes but does not show much change in peak intensity. A similar observation was also made under visible irradiation in Fig. 22(B). The photolysis of 2,4-DCP was tested also without a catalyst under the UV and visible light irradiation at different times various 0 to 80 minutes as shown in Fig. 22(C and D). The peak intensities were slightly decreased under the visible light irradiation when compared to that under UV light irradiation as shown in Fig. 22(D). Thus, the photolysis reaction of MB and 2,4-DCP did not lead to significant degradation under both UV light and visible light irradiation without the catalyst. The calibration plots of MB and 2,4-DCP under UV light and visible light irradiation are shown in Fig. 23(A and B). From these observations for the photolysis reactions, it is evident that the N-ZnONCBs catalyst and light were very important for the photocatalytic degradation of MB and 2,4-DCP (Fig. 24).

## 4. Conclusion

The novel synthesis of N-ZnONCBs catalyst by using a hydrothermal method has been reported and these were synthesized using zinc acetate dehydrate and hydrazine monohydrate as precursors. The absorption spectrum of N-ZnONCBs catalyst was observed in the visible region due to N-doping on the ZnONCBs catalyst. N-ZnONCBs catalyst exhibited the wurtzite phase by using XRD characterization and its average particle size was determined as 61.6 nm and this result correlates with TEM analysis. The successful formation of N-ZnONCBs catalyst was confirmed by FTIR and EDAX. These verified the stretching vibrations of N-ZnO and Zn–O bond in N-ZnONCBs catalyst with FT-IR analysis. N-ZnONCBs catalyst was used as a photocatalyst for the degradation of methylene blue and it showed higher efficiency degradation of MB, as well as pseudo-first-order kinetics under visible light irradiation. The maximum calculated rate of degradation was (*k* = −0.0585 min^−1^ for 100 mg of N-ZnONCBs catalyst) under the visible light irradiation. This catalytic activity was also examined with different dosages, different initial concentrations of MB and different pH 3, 5, 9, and 11 effects under the UV light and visible light irradiation. A zeta-sizer was also used to explore the average size and charge of N-ZnONCBs catalyst. Fluorescence and Quenching studies investigated that the binding interaction between N-ZnONCBs catalyst and MB dye. LC-MS and TOC results clearly determined that N-ZnONCBs catalyst had better photocatalytic activity under visible light irradiation. N-ZnONCBs catalyst was also showed excellent photostability and reusability with a percentage degradation rate of MB (93.2%) after 4 cycle experiments. The colorless pollutants of 2,4-DCP was also degraded with N-ZnONCBs catalyst under visible light irradiation at various time intervals. These results have clearly demonstrated that N-ZnONCBs catalyst can be applied for the photocatalytic degradation of MB from wastewater samples. N-ZnONCBs catalyst was confirmed as a highly stable and reusable photocatalyst for further degradation studies.

## Conflicts of interest

There are no conflicts to declare.

## Supplementary Material
